# Using GIS and stakeholder involvement to innovate marine mammal bycatch risk assessment in data-limited fisheries

**DOI:** 10.1371/journal.pone.0237835

**Published:** 2020-08-20

**Authors:** Gregory M. Verutes, Andrew F. Johnson, Marjolaine Caillat, Louisa S. Ponnampalam, Cindy Peter, Long Vu, Chalatip Junchompoo, Rebecca L. Lewison, Ellen M. Hines

**Affiliations:** 1 Faculty of Political and Social Sciences, Universidade de Santiago de Compostela, Santiago de Compostela, Spain; 2 Campus Do*Mar, International Campus of Excellence, Vigo, Spain; 3 MarFishEco Fisheries Consultants, Edinburgh, United Kingdom; 4 The Lyell Centre, Institute of Life and Earth Sciences, School of Energy, Geoscience, Infrastructure and Society, Heriot-Watt University, Edinburgh, United Kingdom; 5 Environmental Defense Fund, San Francisco, CA, United States of America; 6 The MareCet Research Organization, Shah Alam, Malaysia; 7 Institute of Biodiversity and Environmental Conservation, University Malaysia Sarawak, Sarawak, Malaysia; 8 Vietnam Marine Megafauna Network, Center for Biodiversity Conservation and Endangered Species, Ho Chi Minh, Vietnam; 9 Department of Marine and Coastal Resources, Rayong, Thailand; 10 Department of Biology, San Diego State University, San Diego, CA, United States of America; 11 Estuary & Ocean Science Center, San Francisco State University, Tiburon, CA, United States of America; Maurice Lamontagne Institute, CANADA

## Abstract

Fisheries bycatch has been identified as the greatest threat to marine mammals worldwide. Characterizing the impacts of bycatch on marine mammals is challenging because it is difficult to both observe and quantify, particularly in small-scale fisheries where data on fishing effort and marine mammal abundance and distribution are often limited. The lack of risk frameworks that can integrate and visualize existing data have hindered the ability to describe and quantify bycatch risk. Here, we describe the design of a new geographic information systems tool built specifically for the analysis of bycatch in small-scale fisheries, called Bycatch Risk Assessment (ByRA). Using marine mammals in Malaysia and Vietnam as a test case, we applied ByRA to assess the risks posed to Irrawaddy dolphins (*Orcaella brevirostris)* and dugongs (*Dugong dugon*) by five small-scale fishing gear types (hook and line, nets, longlines, pots and traps, and trawls). ByRA leverages existing data on animal distributions, fisheries effort, and estimates of interaction rates by combining expert knowledge and spatial analyses of existing data to visualize and characterize bycatch risk. By identifying areas of bycatch concern while accounting for uncertainty using graphics, maps and summary tables, we demonstrate the importance of integrating available geospatial data in an accessible format that taps into local knowledge and can be corroborated by and communicated to stakeholders of data-limited fisheries. Our methodological approach aims to meet a critical need of fisheries managers: to identify emergent interaction patterns between fishing gears and marine mammals and support the development of management actions that can lead to sustainable fisheries and mitigate bycatch risk for species of conservation concern.

## 1. Introduction

Small-scale fisheries (SSF) are a critical means of subsistence and livelihood in many regions of the world. They provide needed sources of protein, food security, and poverty alleviation [1, 2], and support the well-being of more than half a billion people worldwide [3]. Despite their importance globally, SSF struggle with sustainability when local communities do not have access to the social capital necessary to participate in resource management [4–6]. As a result, information and data about SSF is often limited as compared to large-scale, industrial fishing operations [7–9]. Furthermore, some SSF have been identified as a threat to marine ecosystems and species [10–12]. Given the tenuous status of many coastal-marine species and the socioeconomic importance of SSF, robust frameworks are needed to support sustainable fisheries and species conservation in SSFss [13–15].

SSF, like other fisheries sectors, incurs fisheries bycatch. Bycatch refers to the unintended capture of non-target species [16, 17], and it has been identified as the largest threat to marine mammals globally [18, 19]. For depleted marine mammal populations, even a few entanglements per year can pose a significant threat [20], especially when combined with cumulative impacts from other anthropogenic threats [21–23]. Bycatch risk is particularly challenging to analyze and calculate in SSF because of data gaps on fishing effort and marine mammal distribution and ranges [9, 19, 24]. Species conservation research developed in close collaboration with local stakeholders, agency personnel, and scientists can be used to overcome these obstacles by characterizing the relationship between SSF and the distribution of threatened marine mammals [25–27].

Recent innovations in geospatial technology have demonstrated success in supporting sustainable fisheries and marine mammal conservation. Global positioning systems coupled with unmanned aerial and marine drones equipped with laser, thermal, and acoustic sensors now enable scientists and conservation practitioners to track marine megafauna movements [28–30] and SSF fishing effort [31], map species distribution and habitat preferences [32, 33] and estimate taxa-specific impacts from human activities [34–36]. In addition, community involvement and local expertise can be integrated with remote sensing and spatial analyses to fill data gaps, characterize uncertainty of existing information, and produce actionable information to address sustainability challenges [37, 38]. Given the growing availability of tools to manage SSF, including those that draw on local knowledge and geographic information systems (GIS), we aim to integrate and visualize existing data on SSF interactions with marine mammals to increase the efficacy of fisheries management research and reduce uncertainty.

A risk assessment evaluates the likelihood, or probability, of an event happening and the magnitude of the consequences if the event happens [39, 40]. Species risk assessment is one approach to support sustainable resource use and conservation by evaluating the risk-reduction potential of different fisheries management options in marine fish stocks, habitats, and ecosystems [41–43]. With a similar goal in mind, geographers and spatial ecologists have developed tools to map and measure the probability of exposure, and resulting vulnerabilities to marine species, from offshore wind farm impacts and vessel noise to fisheries bycatch [44–46]. Studies that use GIS to evaluate risk of these incidental interactions can help address the marine mammal bycatch problem because they present frameworks for the analysis of biodiversity and its susceptibility to one or more threats. Further, spatially explicit marine species risk assessments (e.g., [43, 47, 48]) draw on participatory mapping, spatial analysis and data visualization techniques, which are particularly important in data-limited contexts [38, 49], to engage stakeholders, establish trust, and access local knowledge [50, 51].

Despite advances in GIS technology for data collection, spatial analysis, and risk assessment, there remains a need for tools that incorporate the spatio-temporal dimension of SSF and include both bycatch exposure and its consequences to resident marine mammal populations. For this reason, we developed the Bycatch Risk Assessment (ByRA), a tool for spatially explicit risk assessment tailored specifically for marine mammal bycatch in data-limited fisheries. ByRA combines existing SSF and marine mammal data within an open source GIS-based framework. Most importantly, outputs from the ByRA, which include bycatch risk maps and plots describing species-gear interactions during different seasons and scenarios, are produced in accessible interactive web visualization and summary table formats. These products can therefore easily be communicated to non-expert stakeholders and vetted by experts for iterative improvement that augments the understanding of local fisheries, identifies and fills knowledge gaps, and can be used for designing strategies to meet sustainability objectives [25, 52, 53].

Here, we present the results from an application of ByRA in Southeast Asia, specifically Malaysia and Vietnam. In these countries, fish are a major source of nutrition and livelihoods [54, 55] and managers strive to assemble reliable, accurate, and spatially explicit information about SSF bycatch [19, 25]. The ByRA has two important characteristics that make it useful for understanding the distribution of fishing activities, marine mammals, and their interaction rates over space and time: (1) it is designed for rapid spatial assessment at the site scale, with SSF data inputs and assumptions communicated in a transparent manner; and (2) it is developed in close collaboration with local stakeholders, agency personnel, and scientists, which is critical for actionable evaluation of different bycatch management interventions and strategies. ByRA facilitates stakeholder engagement, identifies areas of bycatch concern, and co-creates knowledge. We describe how ByRA can be used to leverage existing data on animal distributions and fisheries effort, integrate participatory mapping and local expert knowledge within an open source GIS framework.

## 2. Materials and methods

**Fig 1:**
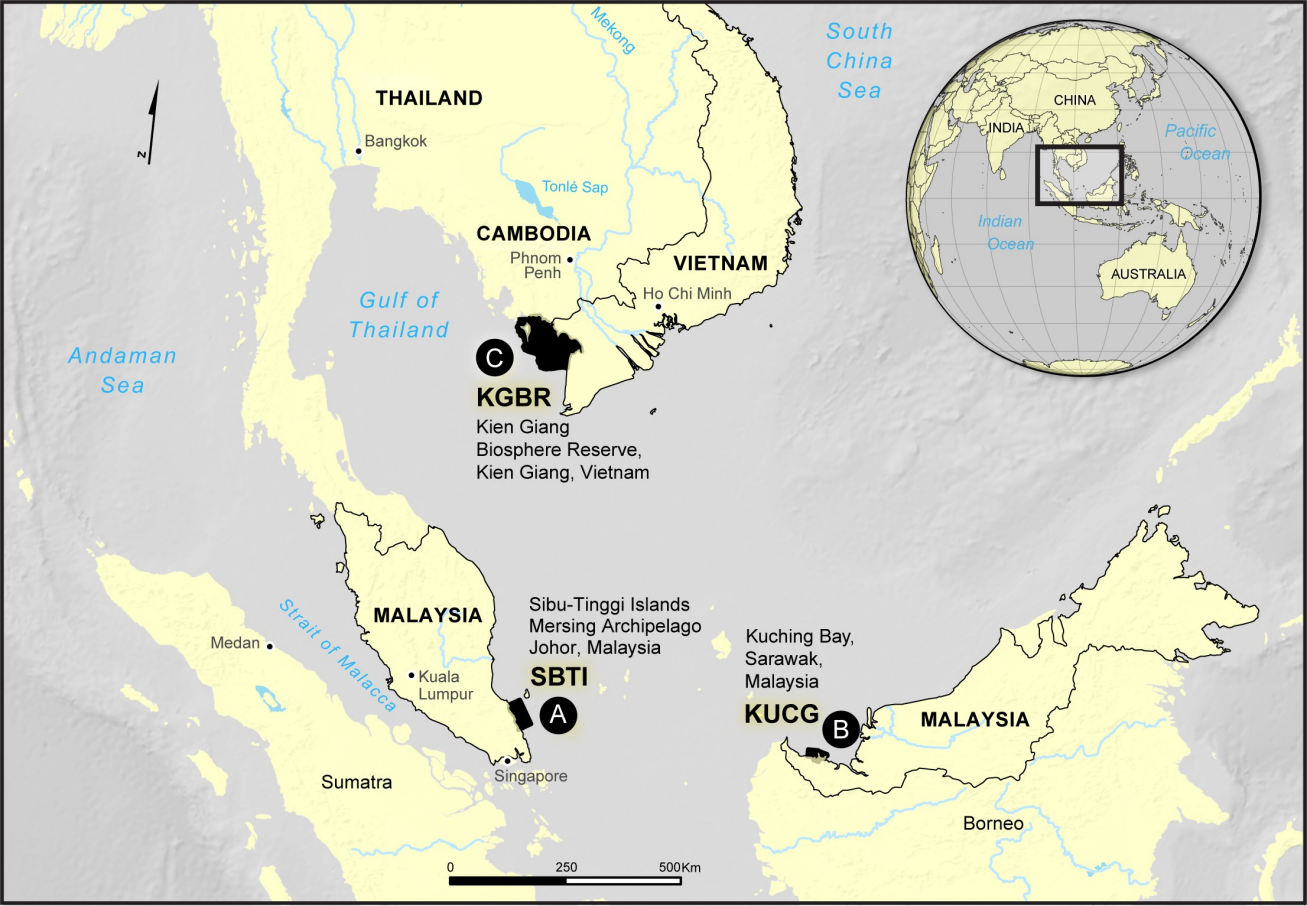
Three field sites selected in Southeast Asia. Areas of bycatch concern shown in black.

To develop the ByRA tool, we used three case study sites in Southeast Asia and the bycatch of two species of marine mammals. The sites were Kien Giang Biosphere Reserve (Vietnam), Kuching Bay and the Mersing Archipelago (Malaysia) (Fig 1). The first two experience bycatch of Irrawaddy dolphins *(Orcaella brevirostris)*, and the latter has a significant dugong *(Dugong dugon)* population. At each of the field sites, we had the support of and worked collaboratively with local researchers and resource management agencies to undertake this work and use their data on marine mammal and small-scale fisheries vessel occurrence.

Bycatch risk was assessed from existing GIS data on marine mammal occurrence and fishing activities. These were mapped as habitat suitability and kernel density estimations, respectively. Fishing data were organized into five general gear categories (hook and line, nets, longlines, pots and traps, and trawls). Drivers of bycatch risk included environmental and sociopolitical factors, specifically seasonal weather (monsoonal) patterns and the current status of fisheries management that influence the distribution of marine mammal populations and SSFs. In Kuching Bay, Malaysia we assessed three scenarios (post monsoon, dry, and pre monsoon) to capture seasonal drivers known to change fishing patterns and Irrawaddy dolphin occurrence over time and, therefore, vary their exposure to bycatch risk, including encounter rates and timing of overlap with different gears in space. In all sites we identified consequences (impacts) of such interactions, including life history stages affected by gear-specific threats and local conservation status of the species. The following sections describe three key steps we took to leverage existing data with the ByRA tool: (i) engage stakeholders and acquire existing knowledge, (ii) build bycatch scenarios, and (iii) analyze and visualize bycatch risk and data uncertainty.

### 2.1. Engage stakeholders and acquire existing knowledge

Our methodological approach emphasized transparency and building collaborative relationships prior to acquiring data, including representatives from provincial governments, non-governmental organizations (NGOs), and scientists from local universities (Table 1). When requesting information, we described our intended use and respected constraints on sharing data based on local management hierarchy, and how it would be reported. For example, we received sensitive information (e.g., animal occurrence) in part because data holders were involved in the project from its inception, understood how the knowledge would be used, and knew their participation would be anonymized through the tool.

**Table 1:** Summary of each field site, including focal species, situational context, and partner(s).

Abbreviated and full name	Focal species	Situational context	Partner(s) and publications
**A) SBTI:** Sibu-Tinggi Islands, Mersing Archipelago, Johor, Malaysia	Dugongs (*Dugong dugon*)	The Mersing Archipelago lies along the southeast coast of Peninsular Malaysia; partners have been studying dugongs and the social science of dugong conservation since 2014	The MareCet Research Organization, Malaysia (Ponnampalam et al. [56])
**B) KUCG:** Kuching Bay, Sarawak, Malaysia	Irrawaddy dolphins (*Orcaella brevirostris*)	Expansive estuarine system near the city of Kuching; partners have been studying cetaceans in Kuching Bay since 2008	Sarawak Dolphin Project; Institute of Biodiversity and Environmental Conservation, Universiti Malaysia Sarawak (Minton et al. [20]; Peter et al. [57])
**C) KGBR:** Kien Giang Biosphere Reserve, Vietnam	Irrawaddy dolphins (*Orcaella brevirostris)*	A Biosphere Reserve designated by UNESCO in 2007; the area experiences one of the highest levels of fishing in the country; the Vietnam Marine Megafauna Network regularly monitors the waters of KGBR	Kien Giang Biosphere Reserve, Vietnam; Southern Institute of Ecology (Vietnam Academy of Science and Technology); Vietnam Marine Megafauna Network (Center for Biodiversity Conservation and Endangered Species) (Vu et al. [58])

The following questions posed during the data acquistion phase yielded the most useful information to perform the ByRA: (i) What kinds of surveys and technology do you use to track marine mammals and fishing vessels? (ii) Which fisheries are present and what fishing gears are used? (iii) Which spatial data are available/exist for your field site to help understand risk of bycatch (e.g., sightings of marine mammals, bathymetric soundings from nautical charts, fisheries management guidelines)? (iv) Does the area have an existing fisheries observer program, stranding network or other indications of work related to marine mammals and, if so, in which season(s) is monitoring conducted?

#### 2.1.1. Areas of interest and subregions

**Fig 2:**
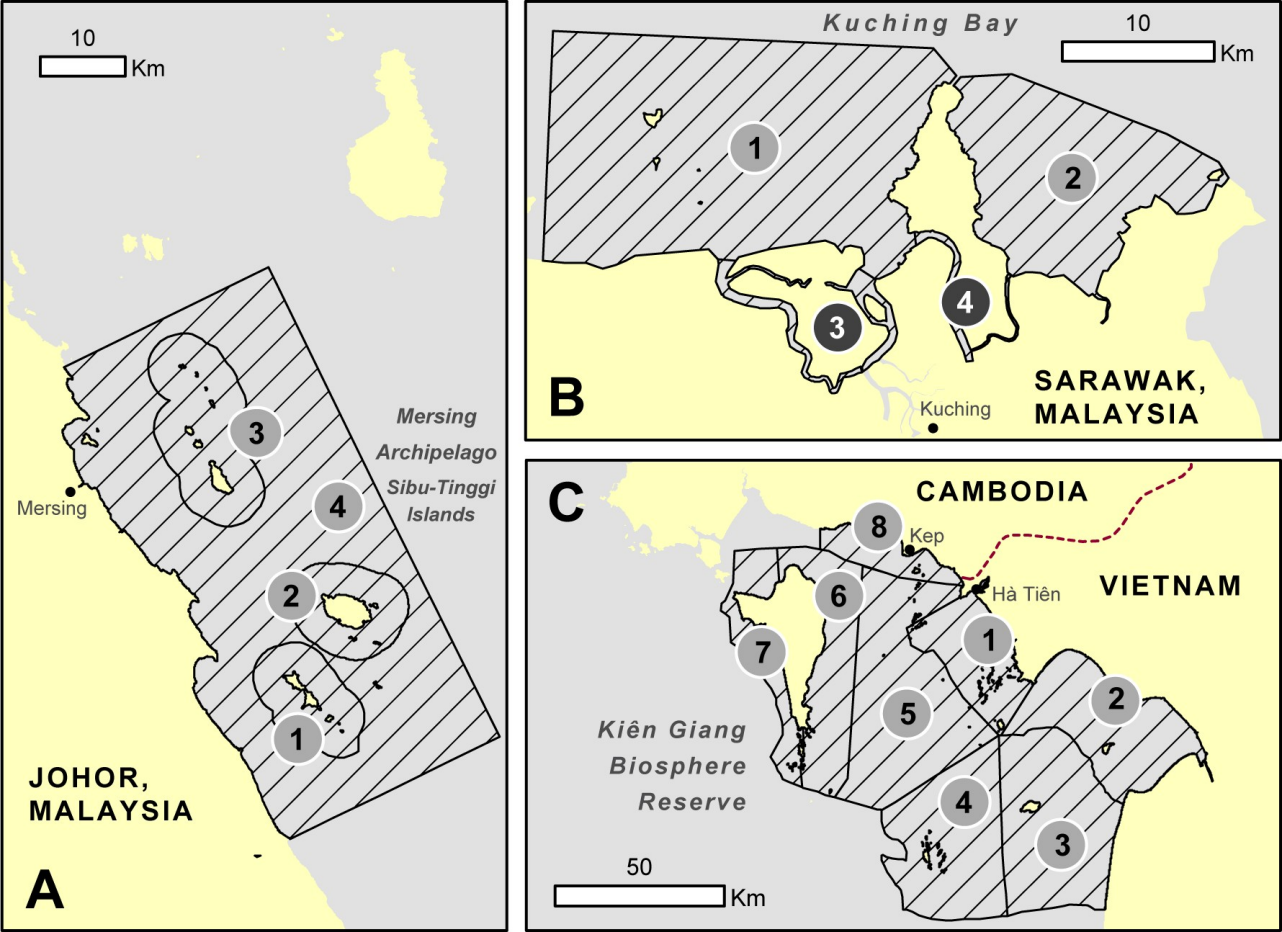
Subregions (numbered circles) based on management, conservation, geopolitical and ecological similarities across the three SE Asian field sites. A) SBTI: Zones 1–3 delineate the 2 nautical mile boundaries of the existing Sultan Iskandar Marine Park. Zone 4 covers the remaining core dugong ranging areas as monitored by The MareCet Research Organization in Johor, Malaysia; (B) KUCG: 1) Santubong-Salak Bay, 2) Bako-Buntal Bay, 3) Salak Telaga Air Rivers, 4) Santubong-Buntal Rivers as in Peter et al. [57]. Darkest grey circles indicate the river network and estuaries of Kuching Bay; (C) KGBR: Zones 1–7 based on survey strata of the Vietnam Marine Megafauna Network.

Based on the spatial coverage of existing marine mammal and fisheries surveys, we structured the risk assessment by delineating areas of interest (AOI) that extended 10 km beyond locations with high SSF and marine mammal occurrence. This minimized edge effects in the geospatial calculations and focused on known areas of bycatch concern. To summarize and compare ByRA findings within each site, we defined between 4 and 8 subregions (Fig 2) that differed by ecological, environmental, and/or governance factors. The AOI for Sibu-Tinggi Islands (SBTI) spanned the existing Sultan Iskandar Marine Park and surrounding waters covering the extent over which the MareCet Research Organization conducts aerial distributional line transect surveys of dugongs and fishing activities [56]. We divided the Kuching Bay site (KUCG) into four subregions as in Peter et al. [57] to capture two ecologically distinct coastal and two hydrologically connected inland areas of Kuching’s expansive riverine system. The AOI for Kien Giang Biosphere Reserve (KGBR) is the entire biosphere reserve, which was subdivided following the survey strata used by the Vietnam Marine Megafauna Network [58].

#### 2.1.2.Fishing activities and gear usage

We acquired information about fishing gears known to entangle, cause strandings or mortality of the two coastal marine mammals of interest. All fishing methods were organized into five broad but distinct categories: (1) nets, (2) trawls, (3) pots and traps, (4) longlines, and (5) hook and line (Table 2). Combining diverse fishing methods into five general gear categories streamlined the data acquisition process by helping local partners identify the most common fishing techniques in each site. For instance, we initially identified more than 20 different gears used by fishers inside KGBR, Vietnam and neighboring Cambodian waters [58]. These five groups of fishing activities known to encounter marine mammals served to elucidate gear-specific impacts during the expert judgement stage.

**Table 2:** Fishing methods and corresponding gear categories identified in each SEA field site.

Gear category	A) SBTI	B) KUCG	C) KGBR
**Nets**	drift net	gillnet (“pukat / ranto”)	anchovy purse seine
			mackerel purse seine
		set net—nylon	purse seine with light
			bottom gillnet
			surface gillnet
	purse seine	drift net (“tangsi”)	shrimp gillnet
			small size trammel net
			sardine gillnet
		trammel net (“pukat 3 lapis / jaring”)	crab gillnet
			crab trammel net
			mosquito net
			set net
	gillnet		
**Trawls**	trawl net	trawl net	single (“normal”) trawl
			pair trawl
			electric trawl
**Pots and traps**	trap	pots and traps	crab trap
			cuttlefish trap
			octopus trap
			rat tail
**Longlines**	bottom line	longline—high: (“rawai timbul”)	fish hooks and lines
		longline—low (“rawai tenggelam”)	
**Hook and line**	line fishing	rod line (“pancing”)	squid hooks and lines

#### 2.1.3. Environmental and marine mammal sightings data

**Fig 3:**
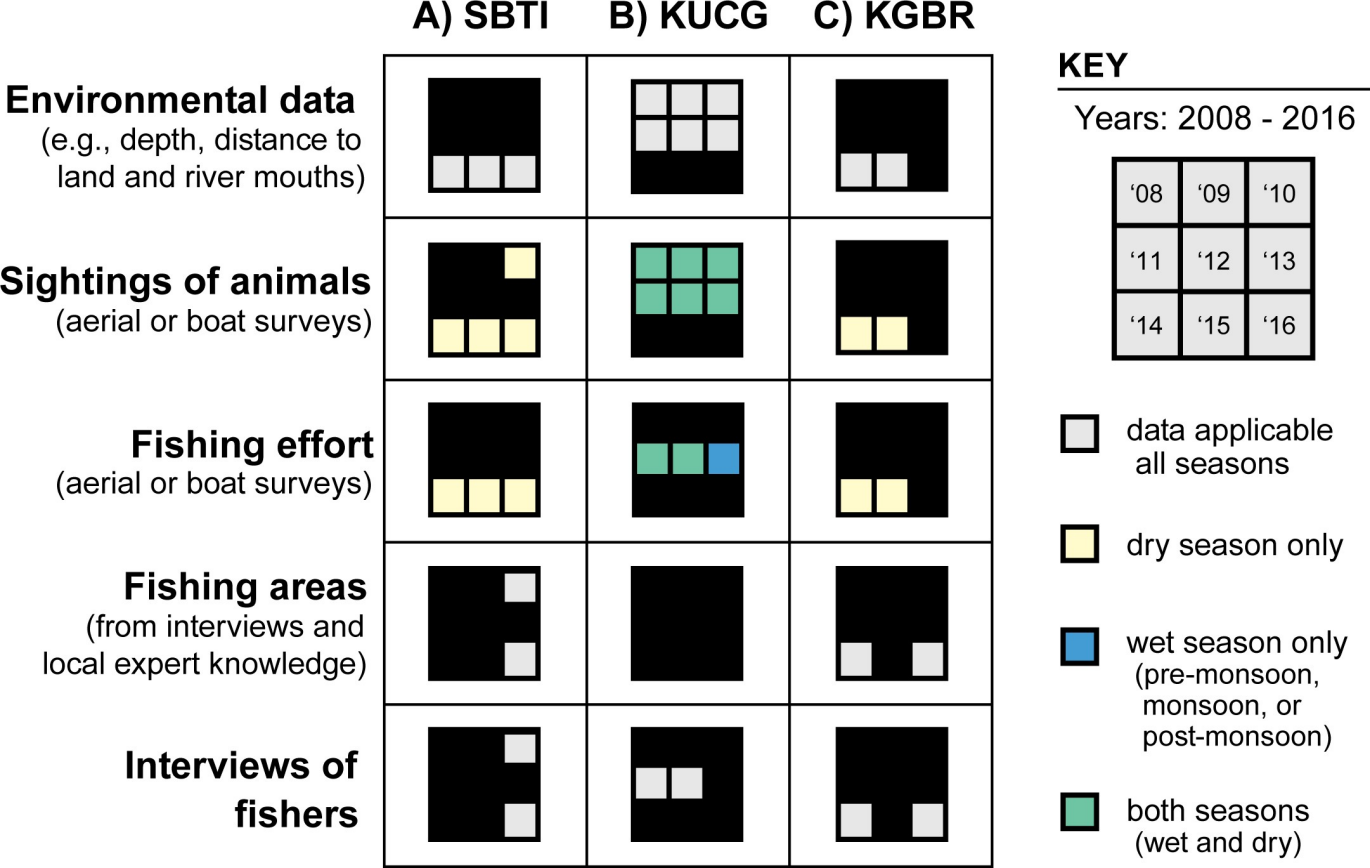
Inventory of environmental, biological, and fisheries data summarized by site, year, and season for each field site. Detailed metadata is available in S1 Table in S1 Data of the supporting information.

We began by compiling globally-available GIS layers (e.g., continental land mass and islands, major rivers, bathymetry) to characterize the coastal-marine environment in the study areas. Three online sources, in particular, (1) Natural Earth (naturalearthdata.com), (2) GADM (gadm.org) and (3) GPS Nautical Charts (fishing-app.gpsnauticalcharts.com) offered free reference layers and viewers to identify available nautical charts for purchase. Monitoring efforts by local partners documented sightings of animals and SSF during aerial and boat-based surveys between 2008 and 2016. These data included GPS location (latitude/longitude), individuals observed (number) or gear type (name), and time of year (season) for each recorded sighting (Fig 3; S1 Table in S1 Data). Drawings of fishing grounds by fishers and government officers were also acquired for the SBTI and KGBR sites.

To prepare these layers for input to ByRA, we leveraged several spatial data processing routines in QGIS, an open source GIS software platform [59]. This included georeferencing and digitizing depth soundings from nautical charts, cost distance analysis, and inverse distance weighting for calculating distance to land and river mouths and producing bathymetric interpolation surfaces. Sightings of marine mammals coupled with environmental data were used in habitat suitability models to estimate their distribution and relative abundance in two Malaysian field sites (see Section 2.2.1. ‘Habitat models’).

### 2.2. Build bycatch scenarios

Scenarios are simplified descriptions of the present and possible futures [60]. In this application of ByRA, we used scenario layers assembled in GIS to highlight suitable habitat areas for marine mammals and the current distribution and intensity of fishing activities by gear type. The scenarios included environmental and socio-political factors such as seasonal monsoons and fishing regulations, e.g., gear restrictions and sensitive habitat zones that can influence the behaviors of fishers and marine mammals. The resulting scenario layers captured emergent patterns of species-gear interactions and were subsequently evaluated in three separate bycatch risk assessments (see Section 2.3: ‘Assess and visualize bycatch risk and data uncertainty’).

#### 2.2.1. Habitat suitability

Habitat models are important tools to link marine mammal observations to environmental variables and identifying critical habitat [61, 62]. To estimate the distribution and relative abundance of dugongs and Irrawaddy dolphins in geographical space, we used species distribution models suitable for small sample sizes [48, 63, 64], or a rule-based GIS approach for habitat suitability designed for data-limited situations. Depth, distance to land, and distance to river mouths have been shown by numerous researchers to be commonly important measures of habitat suitability for dugongs and coastal cetaceans, including Irrawaddy dolphins (see [20, 48, 65]). The selection of the appropriate habitat model, to identify the most important areas within the distribution of a species, is site and dataset-specific [66] and good predictive ability has been achieved with parsimonious models [67].

When marine mammal sightings were available, we applied the Maxent modeling software (biodiversityinformatics.amnh.org/open_source/maxent/) to map suitable environmental conditions. Maxent needs 30 or more sightings for reasonable statistical power [32, 63] and this quantity of occurrence data existed for both Malaysian field sites. Presence-only data of Irrawaddy dolphins and dugongs occurrence were used to quantify the statistical relationship between predictor environmental covariates at locations where a species had been observed versus background locations in which no observation was done [68]. The Maxent algorithm inferred species distribution as a function of relevant environmental covariates [69], which in the SBTI and KUCG sites were water depth (m), seafloor slope (degrees), and/or distance to land and river mouths (km). Next, we converted Maxent outputs from continuous to categorical data in order to match rating scores for each species-gear interaction (see Section: 2.3.2. ‘Spatially explicit criteria’). Habitat suitability layers were reclassified 1 to 3 (lowest to highest suitability) based on the omission rate threshold of 10% (10% of the training occurrence data classified in non-suitable habitats) and the maximum relative occurrence rate (maximum probability for a species to be in a suitable habitat). Maxent variable selection, model testing, performance evaluation and validation is described in the Supporting Information (1.2 ‘Spatial data on species for the spatial overlap criterion’; S2 and S4 Tables in S1 Data; S7, S8, S9 and S10 Figs in S1 Data).

There were insufficient observations of Irrawaddy dolphins (*n = 2*) for a correlative model in Vietnam. Instead, we employed a ‘low-data’ approach to map suitable habitat areas for Irrawaddy dolphins in KGBR based on Briscoe et al. [48], which used a rule-based GIS analysis to designate areas of marine mammal habitat in an area with limited sightings data. The Union tool in QGIS was used to map the overlap between bathymetry and cost distance layers for depicting levels of habitat use in KGBR, specifically: (i) depth range (0–15m), (ii) proximity to major river mouths (<25km) and (iii) proximity to land (<10km) based on previous Irrawaddy dolphin research [20, 65, 70] (S3 Table, S4 Fig in S1 Data).

#### 2.2.2. Seasonality

To account for changes in fishing activity and Irrawaddy dolphin habitat use throughout the year, we defined seasonal scenarios in KUCG and analyzed species-gear interactions over three distinct periods of time–i.e., post-monsoon (March to May), dry season (May to September), and pre-monsoon (September to December). In SBTI, aerial surveys to monitor dugongs and SSF around the Sibu-Tinggi Islands were conducted during the dry season only, typically from November to February, to avoid the northeast monsoon [71]. Fishing activity is less intense during the wet season (Lee S.F., personal communication, January 25, 2017) and so we focused on estimating dugong bycatch risk between March and November. In KGBR, an annual composite was used to identify spatial patterns of bycatch risk, as SSF activities remain relatively constant throughout the year in Kien Giang, Vietnam.

#### 2.2.3. Fishing extent and intensity

To map fishing intensity by gear type, we used kernel density estimation (KDE), an interpolation technique available in QGIS for mapping hot spots that estimates location, spatial extent, and intensity of fishing activity [72]. This non-parametric kernel method uses the probability density function of a random variable (in this case, fishing gear incidence) and fits a smoothly tapered surface to each point [73]. A limited search distance parameter (1 or 2 km) was applied based on the distance between each boat or aerial line transect to create a continuous surface that represented the relative magnitude of fishing intensity by gear type over the entire area of interest (S11 Fig in S1 Data). KDE was applied in two Malaysian field sites to analyze the gear occurrence data, collected as individual point locations. In KGBR, we compiled map layers representing fishing grounds based on areas previously identified during fisher interviews and by provincial government staff. Due to limited sightings of fishing activities in southern Vietnam, the “intensity of gear use” exposure criterion was omitted from the risk equation.

### 2.3. Assess and visualize bycatch risk and data uncertainty

**Fig 4:**
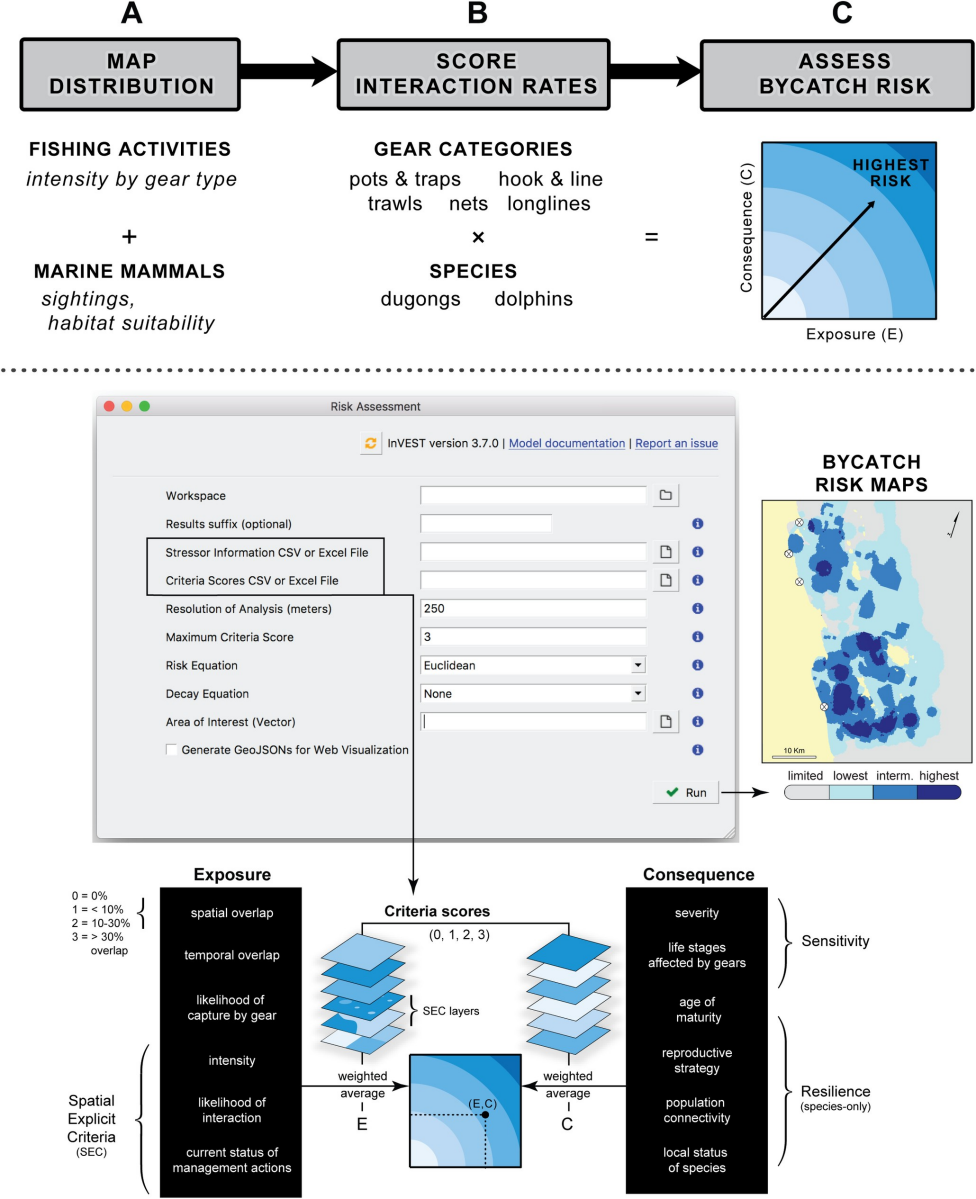
Bycatch risk assessment conceptual model and tool process diagram. Top panel depicts how layers and rating scores are assembled and combined. Bottom panel shows tool interface and steps to estimate risk for each grid cell within an area of interest. Colored bands in the risk plot are numerically determined and based on the range of exposure and consequence scores (0, 1, 2 and 3 in this assessment).

To assess risk of bycatch in each site, we combined geospatial layers of (1) species distribution, based on habitat suitability, and (2) fisheries presence, organized by gear type, distribution, and intensity of use (Fig 4A). The core functionality of the ByRA tool–to draw on assembled scores of interaction rates and assess bycatch risk (Fig 4B and 4C)–is executed through the user interface of InVEST, a freely downloadable software suite (naturalcapitalproject.stanford.edu/software/invest) [74]. Here, we adapted the exposure-consequence criteria for habitat risk assessment [47, 75], where risk of fisheries bycatch is calculated as a function of the likelihood of *exposure* (interaction between the marine mammal and the fishery), and its *consequence*, which is the gear-specific impact to a species. For two additional exposure criteria unique to bycatch risk, we defined: (1) *likelihood of interaction*, as the probability that the animal will encounter a fishing gear if spatial overlap was detected, and (2) *catchability*, as the likelihood of animal capture by a gear type when this overlap occurs. Similar to Samhouri and Levin [43], species-only *consequence* attributes were defined as: (a) the resilience of a species to a stressor (based on age of maturity, reproductive strategy, population connectivity, local status of species) and (b) its sensitivity (mortality and life stages affected by gear).

#### 2.3.1. Expert evaluation

Species-gear interactions for a total of twelve bycatch exposure and consequence criteria were scored as guided by field observations, literature review, and, subsequently, expert opinion (Table 3). In August 2017, researchers and agency personnel specializing in marine biology, fisheries ecology, marine veterinary medicine, biogeography, and social sciences participated in a judgment process to score interactions (1 to 3, low to high contribution to risk) along with their confidence in each opinion. Representatives from each field site, with working knowledge of marine mammals and SSF activities, set the final rating scores. The supplementary information lists individual exposure (E) and consequence (C) criteria, along with justifications for the interaction ratings, data quality and attribute weights (S5 and S6 Tables in S1 Data).

**Table 3:** Definitions and scoring bins for the exposure and consequence criteria and Spatially Explicit Criteria (SEC).

Criteria	High risk (3)	Medium risk (2)	Low risk (1)	Description
***Exposure (likelihood) criteria***				
**Spatial overlap**	>30% of species overlaps with gear	10–30% of species overlaps with gear	<10% of species overlaps with gear	The overlap by grid cell between the distribution in space of each species and gear is calculated by the toolbox.
**Temporal overlap**	all year (12 months)	most of year (4–11 mo.)	occasional (1–3 mo.)	The duration of time that the species and gear overlap in space.
**Intensity of gear use**	high intensity	medium intensity	low intensity	Overlap between gear-type density and species distribution. (SEC)
**Likelihood of interaction between gear and species**	high likelihood	medium likelihood	low likelihood	The overlap between habitat suitability and intensity of gear use. The resulting encounter rates are ranked low to high. (SEC)
**Likelihood of capture by gear**	high likelihood	medium likelihood	low likelihood	The “catchability” of species by gear includes behavior of animal during interaction, for example, dugong may roll around nets.
**Current status of management**	no strategies identified / implemented	management strategies identified, not implemented	management strategies identified & implemented	Management strategies can limit the use of certain gears in certain areas, thereby mitigating negative impacts to species. (SEC)
***Consequence (impact) criteria–******sensitivity***				
**Mortality**	lethal	sub-lethal	negligible	The severity (direct effect) of gear on mortality rate of a species.
**Life stages affected by gear**	adults only	mixed	juveniles only	If a gear strands a species before they have the opportunity to reproduce, recovery is likely to be inhibited.
***Consequence (impact) criteria–******resilience***				
**Age of maturity**	> 4 years	2–4 years	< 2 years	Greater age at maturity corresponds to lower productivity.
**Reproductive strategy**	long calving interval / high parental invest	medium calving interval / high parental invest	short calving interval / med parental invest	The extent to which a species protects and nourishes its offspring.
**Population connectivity** (DPS = distinct population segment; ESU = evolutionary significant unit)	negligible exchange between the focal regional population and other populations	occasional movement/ exchange between the focal regional population and other populations	regular movement/ exchange between the focal regional population and other populations	The realized exchange with other populations based on spatial patchiness of distribution, degree of isolation, and potential dispersal capability; based on monitoring surveys or direct tracking estimates. 3 = DPS or ESU; 1 = not a DPS or ESU
**Local status of the species**	endangered	threatened or of concern	low concern	The conservation status of the species in-country.

#### 2.3.2. Spatially explicit criteria

When available GIS data could be used to characterize species-gear interaction rates, spatially explicit criteria (SEC) layers were created to differentiate rating scores over space (1 to 3, lowest to highest exposure or consequence, Fig 4). For this application of ByRA we mapped and scored interaction rates for three exposure criteria: (i) *intensity of gear use*, (ii) *current status of management*, and (iii) *likelihood of interaction between the gear and species* (Table 3, S11, S12 and S13 Figs in S1 Data). A geospatial workflow was coded as a plugin for QGIS (available at mmbycatchtoolbox.org) to automate the necessary GIS operations (i.e. unions and definition queries) for preparing ByRA SEC input layers. Each output was reclassified (1 to 3; low to high) using a Jenks natural breaks algorithm to minimize the variation within each class. Encounter rates, or ‘likelihood of interaction’, were calculated as the sum of overlapping layers for habitat suitability (1–3) and gear intensity (1–3), where a sum total of 6 or 5 = high, 4 = medium, and 3 or 2 = low likelihood of interaction between gear and species. Current status of management was scored as “1” if implemented and “2” if identified for a given area. Areas where no management or regulation was identified were scored “3”, which was the maximum score (highest contribution to exposure) for this criterion.

#### 2.3.3. Measuring bycatch risk

Two common methods for measuring environmental risk based on expert opinion are Euclidean distance and multiplicative functions. Cumulative impact mapping studies tend to use a multiplicative approach [76, 77], whereas species risk assessments typically estimate risk as the Euclidean (straight-line) distance for each species-threat combination in risk plots [43, 47], which leads to a more precautionary scoring and higher risk [39, 41]. A recent evaluation of qualitative risk assessment frameworks suggests better model performance using a Euclidean distance measure [78]. Therefore, we selected Euclidean distance, from the origin (minimum score) to the average of criteria scores for exposure (*E*) and consequence (*C*), to quantify bycatch risk (Eq 1). If a stressor and species did not overlap, the tool assumed that *E* = 0, *C* = 0, and therefore Risk (*R_ij_*) = 0 for the grid cell being evaluated.

$$R_{ij}=\sqrt{{\left(E-1\right)}^{2}+{\left(C-1\right)}^{2}}$$ (1)

#### 2.3.4. Characterizing uncertainty of data sources

We applied a variable weighting structure and data quality scores–i.e., a weighted-average where *d_i_* = data quality weight and *w_i_* = attribute weight–to account for data uncertainty and substantiate the species-gear interaction ratings for each site (Eq 2, S7 Table in S1 Data). To characterize data input uncertainty for stakedholder outreach, we designed a simple tri-color matrix (Table 4). ByRA outputs coupled with a visualization of data quality were shared with managers to convey how existing information in data-limited sites could be used to further improve the quality of risk estimates over time.

$$E=\frac{\sum_{i=1}^{n}\frac{e_{i}}{d_{i}∙w_{i}}}{\sum_{i=1}^{n}\frac{1}{d_{i}∙w_{i}}}$$

$$C=\frac{\sum_{i=1}^{n}\frac{c_{i}}{d_{i}∙w_{i}}}{\sum_{i=1}^{n}\frac{1}{d_{i}∙w_{i}}}$$ (2)

**Table 4:** Diagnostic to characterize data uncertainty based on where existing information fits along a spectrum of green-yellow-red (highest to lowest data quality, respectively).

Data type	Green	Yellow	Red
Animal sightings distribution	Data collected during line transect survey and could be used to estimate relative abundance with robust methodologies and measurements of uncertainties.	Sightings/photo id collected during opportunistic surveys; relative abundance estimation might be possible.	Very few sightings collected during line transect or opportunistic survey; no formal abundance estimation possible.
Habitat suitability	Estimated from modeling; quantification of uncertainty available from modeling and collection of environmental variables.	Estimated using non-modeled distribution methodology; minimal environmental variables collected.	Information from other regions used to estimate animal distribution.
Fishing effort / gear type densities	Data available such as fishing effort per unit of distance or time possible using modeling; uncertainty measurements possible.	Spatial distribution of fishing gears, relative (to time or space) fishing effort or fishing gears, based on interviews or expert opinion.	Sparse or incomplete data; no geospatial or precise localization of the fishing effort/gear distribution.
Bycatch / stranding data	Robust data about bycatch available from interviews, boat survey, or stranding records; estimation of bycatch rate possible along with measurement of uncertainties.	Relative estimation of bycatch from interviews or stranding data.	No estimation of bycatch or strandings available.

## 3. Results

ByRA generated accessible, non-technical maps for visualizing bycatch risk estimates. Map outputs captured spatial trends in species distribution and fishing effort to highlight fishing areas likely to have high interaction rates as well as seasonal changes in bycatch risk. The uncertainty of data inputs was characterized and outputs were error checked and improved by local experts and stakeholders.

### 3.1. Visualizing bycatch risk

**Fig 5:**
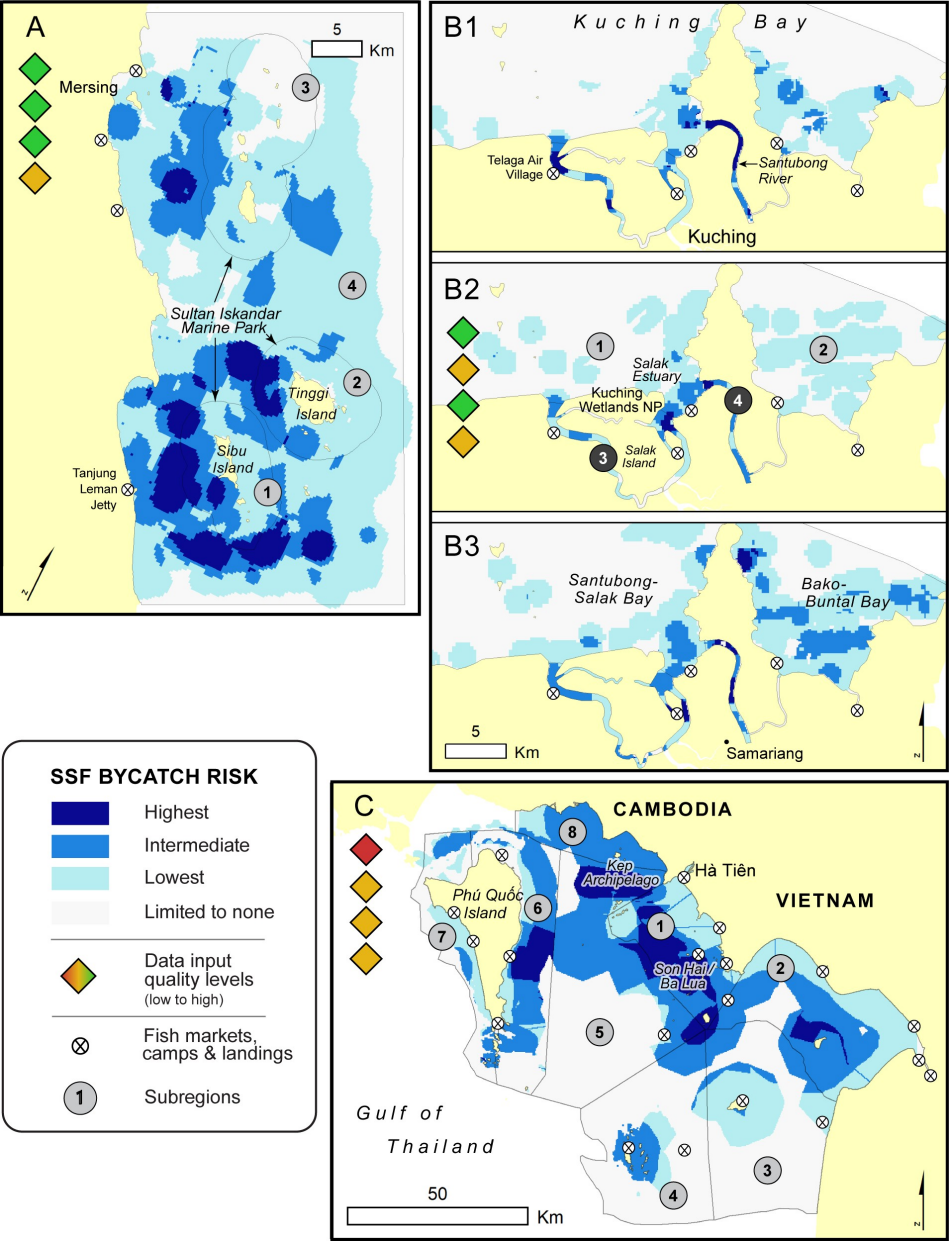
Estimated bycatch risk in three field sites (A-C). (A) Sibu-Tinggi Islands, Johor, Malaysia (SBTI) for dugongs, (B) Kuching Bay, Malaysia (KUCG) during the dry season, pre-monsoon and post-monsoon, B1-3 respectively, and (C) Kien Giang Biosphere Reserve, Vietnam (KGBR) for Irrawaddy dolphins. Data quality levels of four categories of ByRA inputs from Tables 4 and 5 are displayed as colored diamonds.

We found that risk estimates in ByRA were driven primarily by the fishing method (gear type) and the density of fishing activities that were found to overlap suitable marine mammal habitat areas (“intensity of use” and “likelihood of interaction” exposure criteria, respectively). Areas with high marine mammal occurrence and fisheries activity were predicted as highest risk of SSF bycatch. Specific to the three field sites, areas where nets and trawls were used (gears rated as highest likelihood and impact on a number of exposure and consequence attributes by local experts and the literature, S5 and S6 Tables in S1 Data) were identified by the tool to pose substantial risk to both Irrawaddy dolphins and dugongs. By season and scenario, a range of bycatch risk maps were produced–classified as lowest, intermediate and highest risk–the latter of which served to pinpoint areas of greatest bycatch concern (Fig 5).

**Table 5:** Classification of data input uncertainty in three SEA field sites. Green = substantial data available, yellow = limited data available, and red = data are either incomplete or severely limited.

Data type	Field sites		
	SIBU	KUCG	KGBR
Animal sightings distribution	Systematic transect aerial survey	Systematic transect boat survey	Systematic line transect boat survey; not enough sightings to characterize distribution
Habitat suitability	Seagrass data and mammal acoustics; limited environmental data collected during survey	Environmental data collected with the transect survey	Environmental data partially collected
Fishing effort / gear type densities	Collected during line transect surveys and from interviews		From interviews only
Bycatch / stranding data	From interviews and some records of stranded animals due to interactions with fisheries	Presence/absence of bycatch from interviews only	

**Fig 6:**
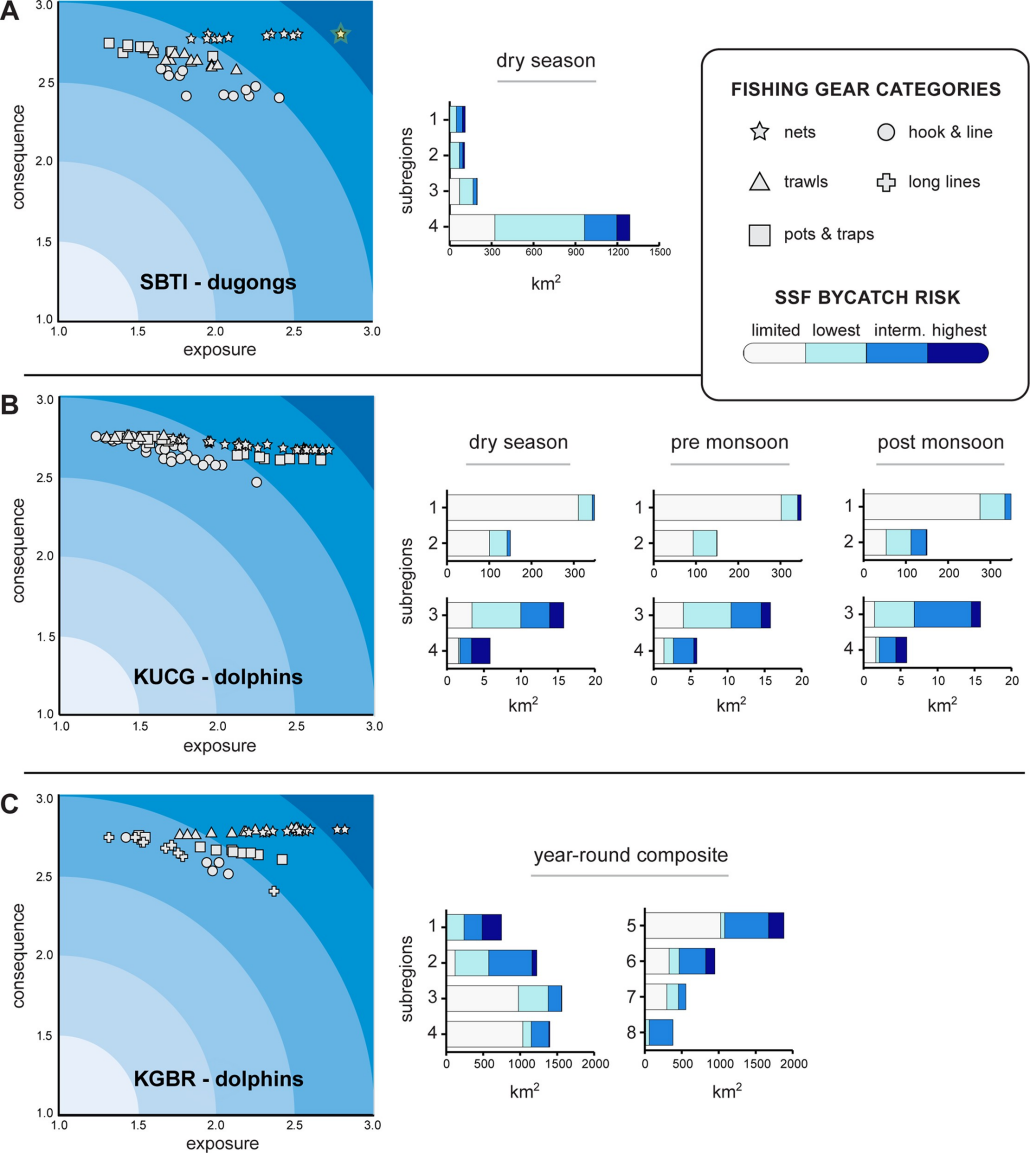
Plots and bar charts summarizing drivers and emergent patterns of bycatch risk. Coordinates (grey symbols) mapped as the weighted average of exposure and consequence criteria scores. They explain the contribution to risk of each gear category by subregion, habitat suitability type, and scenario. ByRA calculates risk based on distance from the origin (exposure = 1.0, consequence = 1.0) to each coordinate on a cell-by-cell basis where darker blue indicates higher risk. Star symbol with yellow halo at the top-right indicates conditions of highest risk. Bar charts show total area at risk (x-axis) by risk level (blue color palette) and subregion (y-axis) for each field site (A-C) and scenario. Note the two x-axes for KUCG site in panel B.

Visualization of risk outputs also identified drivers of risk by gear type and subregion (Fig 6). Nets and trawls were scored by local experts to have a considerably higher likelihood (exposure) and impact (consequence) to both marine mammal species where they co-occurred, while pots and traps were more benign, especially for dugongs. The top-right corner of ByRA risk plots (darkest blue color bands in Fig 6) indicated which gears were the strongest drivers of bycatch risk, when each species-gear interaction occurred. This included nets for dugongs in SBTI, nets and pots and traps for Irrawaddy dolphins in KUCG, and additionally trawls for Irrawaddy dolphins in KGBR. If these interactions were to occur in areas of highest suitability for marine mammals, the estimated risk increased further (rightward movement along the x-axis) due to a greater likelihood of species-gear interaction and, therefore, higher average exposure score. Variation in bycatch exposure over space and time was captured as gear-specific exposure ratings, and then reflected as a subset of spatially explicit criteria input layers (Fig 6, S11-13 Figs in S1 Data). Separately, we shared SEC layers in a simple visual format (maps and tables) with marine mammal scientists and managers to facilitate discussions about data uncertainty and validate preliminary findings.

## 3.2. Capturing expert knowledge

ByRA’s map input layers and species-gear interaction scores were iteratively improved through expert review and feedback using interactive maps and discourse in a participatory GIS framework. A combination of regional meetings, workshops, and expert interviews served to refine the approach and confirm early results. A transparent and flexible approach to stakeholder involvement and risk assessment was cited as key by our collaborators to build trust in the process and elicit local knowledge often buried in reports and on the data hard drives of representatives from provincial governments and other institutions. For example, colleagues based in Vietnam, who were not able to attend a meeting with our team, later provided key data layers on the location of river mouths for modeling Irrawaddy dolphin habitat suitability and common gears observed by government officials in fishing areas where GPS use was prohibited.

Two site visits in 2017 served to build a shared understanding of the ByRA approach among the project team and how ByRA could be further standardized to accommodate varying quantities and qualities of data and fill critical information gaps,. The second site visit was a ByRA learning exchange workshop with our in-country collaborators to demonstrate how they could apply the tool in their home countries. We found that including these face-to-face meetings in our project budget was a necessary step to galvanize action across the region in support of research efforts, specifically, to make better use of existing data from animal and SSF observation records, expert knowledge, government reports, and the literature. These connections also made subsequent engagement and remote support for applying the ByRA in new geographies easier because many scientific and technological hurdles (e.g., capacity strengthening, understanding the methodological approach, user interface and data uncertainty) had been overcome.

## 3.3. Characterizing uncertainty

In-person meetings with stakeholders in August 2017 yielded a diagnostic for data uncertainty, showing a gradient of data input quality across the three field sites (Table 5). This was used by our team to identify and discuss commonalities across locations and taxa, prioritize new techniques to undertake such as local surveys of environmental data, animal occurrence, fisheries effort, and acknowledge uncertainty as part of outreach. We found that by visualizing uncertainty of data inputs qualitatively (using green, yellow and red colors of a globally-recognized traffic light signal), scientists and managers had a clearer set of priorities for future acquisition and integration of existing information; with an emphasis on filling data gaps, reducing data uncertainty in areas of highest bycatch concern identified by the tool, and restarting monitoring activities that had stalled due to funding limitations.

ByRA map outputs were also error checked by in-country collaborators with certain areas flagged as potential over- or underestimates of fishing activity and habitat suitability, two important drivers of bycatch risk in the tool. We could only characterize data uncertainty and validate outputs because local experts were present to corroborate bycatch risk estimates based on information previously provided by researchers and knowledge holders. The visualization of uncertainty across a range of data inputs and field sites assisted researchers in Malaysia, Vietnam, and Cambodia to chart a path forward by concentrating limited resources in areas with substantial information gaps and consider monitoring protocols and technologies that had already yielded substantial returns in neighboring regions (see Vu et al. [57] for KGBR Vietnam example).

## 3.4. Emergent patterns and findings

For all three SEA field sites, ByRA outputs identified emergent spatial trends of interactions between SSF and marine mammals; specifically, fishing gears and locations that are likely to have high bycatch rates and damaging effects on marine mammal population viability. For instance, coastal areas with the highest level of bycatch risk to dugongs (darkest blue bars in Fig 6A) were well distributed across the four subregions of SBTI (between 13–44 km^2^) despite subregion 4 being the most expansive (68% of combined total area of subregions 1–3), closest to the main fishing village (Mersing), and least protected (no fisheries management identified). ByRA revealed this and other non-obvious patterns that would be difficult to uncover without a spatio-temporally explicit risk assessment framework. For instance, risk coordinates plotted for each species-gear interaction and range of habitat suitability highlighted that nets deployed in some areas of SBTI posed an intermediate level of bycatch risk, similar to other SSF gears. On the other hand, risk from nets was highest inside subregion 3 (Figs 5A and 6A). Furthermore, area summaries of bycatch risk levels as bar charts (Fig 6) displayed how risk shrinks and expands over space and time, including seasonal changes in total area of estuarine and coastal waters at greatest SSF bycatch risk.

As illustrated in the Kuching Bay (KUCG) field site, seasonal snapshots depicted how bycatch risk is likely to change during the year and within subregions (Fig 5B). For the dry season (May to September) the greatest proportion of intermediate-highest risk, relative to total area, was inside the river system of subregions 3 and 4 (37 and 70%, respectively) compared to just 2 and 5% in coastal subregions. After the wet season, additional risk hotspots emerged in coastal areas of subregions 1 and 2 (Fig 6B) because SSF activities increased and Irrawaddy dolphin occurrence was high in these areas. Distance to land was identified by Maxent to be the most important environmental variable for Irrawaddy dolphins post-monsoon (50% overall contribution; S4 Table in S1 Data). As a result, bycatch risk estimates shifted to their highest levels in these coastal areas of KUCG until the dry season, specifically where there was high SSF occurrence and no fisheries management strategies identified.

The most data-scarce of the three SEA sites, KGBR Vietnam represents a template for ByRA users applying the tool in places where existing data are severely limited (red to yellow uncertainty levels in Table 4). Despite relying entirely on data collected by others who used indirect measures of SSF activity and marine mammal distribution (i.e. fisher interviews, participatory mapping, and environmental overlays such as distance to land, depth and other optimal habitat variable ranges), we found three distinct areas within subregions 1, 5 and 6 that accounted for almost all (88%) of the highest level of bycatch risk across the KGBR site. Therefore, it was still possible to identify specific locations in southern Vietnam–i.e. greatest likelihood of interaction between dolphins and high-impact gears (nets and trawls, in this case)–as priority candidates for further monitoring and data collection.

## 4. Discussion

In this study, we present spatially explicit estimates of bycatch risk in three SE Asian field sites. A total of 10 810 km^2^ of estuarine and coastal waters were systematically screened to home in on 805 km^2^ (approximately 7.5% of the total area of interest) identified as highest level of bycatch risk to dugongs and dolphins. For these areas of concern, nets and trawls were the gear types associated with the highest bycatch risks in large part due to greater exposure (distribution and intensity relative to other SSF gears) and consequences (mortality) when these fisheries encounter marine mammals. The spatially and temporally explicit scenarios in Kuching Bay showed patterns of risk that shifted to and from the estuarine and coastal waters across seasons. By integrating information from fisher interviews and line-transect surveys, we mapped the likelihood of species-gear interactions over space and time and at local scales. In parallel, geospatial analysis techniques such as participatory mapping with local scientists and agency experts were used to build habitat suitability layers by site and season. Finally, SSF gear and species interaction rates were scored to assess and map bycatch risk and data uncertainty. These ByRA outputs demonstrated the potential of a new tool to co-create knowledge and garner insights about bycatch exposure in data-limited small-scale fisheries.

Characterizing the effects of marine mammal bycatch through space and time can be compared to finding a needle in a haystack–as bycatch is difficult to observe and quantify [18, 79]. However, the results from ByRA are encouraging. Despite the challenges to sustainability that small-scale fisheries face, ByRA demonstrates the ability to make use of limited data as a means of addressing some of these challenges, e.g., pinpointing fishing areas where and when to concentrate efforts (monitoring, education, and outreach) and, conversely, identifying low risk areas where additional effort is not warranted, saving time and resources. This kind of information is urgently needed in Southeast Asia, and many parts of the developing world, where resources and capacity to conserve marine biodiversity and mitigate bycatch are scarce [6, 25]. This can also help fisheries in developing countries comply with new import regulations from provisions within the Marine Mammal Protection Act [14, 15].

Against a backdrop of the importance of SSF as nutrition source and livelihood for coastal communities [3, 80], effective bycatch mitigation depends first on identifying emergent patterns of exposure, which is driven by myriad factors including prey abundance, seasonality, and gear preferences [36, 81, 82]. Nevertheless, outputs from ByRA identified patterns in bycatch occurrence (e.g., particular species, fishing gears, and locations) that had high interaction rates. For instance, in subregion 3 of SBTI, interactions between nets and dugongs were numerically determined to be the highest risk level (2.79 for exposure and 2.80 for consequence; max score of 3.0) of all species-gear interactions evaluated. Interestingly, the bycatch exposure score was highest for dolphins in KUCG with nets deployed during the pre-monsoon in subregion 4 (2.71 out of 3.0), which aligns with evidence that gillnets are an acute threat causing direct mortalities to marine mammals in significant numbers worldwide [18, 19]. By disentangling this and other drivers of exposure, we found patterns of highest bycatch risk under conditions of (1) high species-fishery encounter rates, (2) high-impact gears in use (especially nets and trawls) (3) no management identified, and (4) suitable marine mammal habitat.

### 4.1. Limitations and simplifications

An obvious information gap in our study was bycatch data from onboard observers, a common requirement in European and US fisheries policy [83, 84], and a monitoring technique in SEA that is not commonly utilized. To date, efforts to characterize bycatch and map risk in Southeast Asia have relied almost entirely on fisher interviews to map the extent of fisheries operating in the region [48, 85]. Without technology to comprehensively monitor use of the marine environment by fishers, we were unable to capture activities or interactions that occur at night or with discarded or unattended gear. The use of onboard observers, remote electronic monitoring (REM), and other rapid, low-cost technologies [85], would greatly enhance the ability of ByRA to identify high-risk, under-surveyed areas. However, one of the strengths of the ByRA approach is the ability to accurately described and account for data uncertainty, a clear demonstration of how even low-resolution information can be applied to more effectively investigate risk and guide future bycatch monitoring and management [79, 86]. For example, areas of highest bycatch risk in the SBTI field site provide more evidence that supports recent calls for designation of a dugong sanctuary inside the Mersing Archipelago [56]. Likewise, despite a paucity of data in KGBR, habitat suitability maps derived entirely from GIS overlays showed strong agreement with Irrawaddy dolphin sightings acquired independently of the ByRA analysis (S4 Fig in S1 Data) [58, 87]. Although these analyses provide more information and insight on bycatch risk areas and management interventions that are likely to reduce risk, additional research is needed to compare modeled outputs to other modeled empirical data on bycatch rates, or strandings [88, 89].

Another consideration for future research is the need to account for antagonistic or synergistic effects, that may better reflect the total risk of bycatch to the species [78]. While ByRA’s default calculation of cumulative risk is additive (sum of individual risk scores for all gear types evaluated), intermediate outputs can be reanalyzed and combined as appropriate in each decision context. There is also a need for better data on the interaction rates of species with SSF gears such as gillnets and traps that also have ecological impacts to habitats and ecosystems [36, 82]. We took a precautionary approach of maximum risk based on experts opinion (as in [39, 41, 81]), due to the strong evidence that associates SSF gear with marine mammals stranding and mortality [21, 22]. Data uncertainty with fishing locations are also an important consideration. Our kernel density estimates were limited by a survey line artifact (circular horseshoe pattern), that when reclassified into three levels of exposure gave the effect of discontinuity in the fishing effort and gear-type intensity maps (S11 and S13 Figs in S1 Data). Data uncertainty levels yellow and red (adequate to limited data quality) served to flag this and other areas for improvement (Table 5).

### 4.2. Future directions

Marine mammal scientists and conservation groups in the SEA region continue to collect data on environmental habitat variables to understand seasonality, cetacean behaviors, and enhance the effectiveness of protection and management measures [33, 56]. This information is critical, especially to reduce bycatch risk. These data will inform habitat model selection [32, 57, 90] and increase ByRA’s analytical complexity [79] in support of more dynamic ocean management [35]. For example, marine megafauna networks in Vietnam and Cambodia aim to fill information gaps over the next few years, e.g., additional sighting records of Irrawaddy dolphins for correlative habitat models such as Maxent, while integrating local knowledge and strengthening capacity to generate actionable information for communicating with government officials and policy makers over the longer term [58]. These efforts may be more feasible in areas identified by the tool as highest relative risk (e.g., subregions 1–2 and 5–6 in KGBR) and where there is likely to be interest in the conservation of important marine mammals for tourism and alternative livelihoods [4, 5, 80]. Deployment of other technologies such as passive acoustic monitoring and telemetry can also aid in these efforts.

Species-gear interactions and their impacts vary widely by location and across small-scale fisheries [25, 36, 82], which underscores the importance of ByRA as a tool to integrate existing knowledge, characterize bycatch likelihood and identify areas where bycatch risk is high. We found that a substantial investment in the process of scoring species-gear interactions (exposure and consequence criteria) based on available field data, literature, and expert knowledge was essential to capture salient effects associated with small-scale fisheries gears and other local fishing methods. In the SEA case studies, consequence criteria scores had a limited range because only one species was evaluated in each site. Still, variation in the final weighted average of exposure criteria scores highlight how much risk estimates posed by one gear can vary over space and time (e.g., 1.71 to 2.81 range of exposure scores for nets within SBTI). Spatial planners and managers can benefit from this insight by mapping fishing gears and at-risk marine species [46, 65, 91] and then applying ByRA to identify bycatch hotspots where mitigation is needed to reduce bycatch risk.

There are also opportunities to apply ByRA for multispecies assessment, which can illuminate high risk gears across species by season or scenario. This may include comparing risk between fishing areas, how different gears contribute to risk, or evaluating alternative management strategies under consideration [43, 47, 92]. Through leveraging global systems and regional seafood ratings programs that compile small-scale fisheries knowledge [9, 93], multi-species risk assessment can be applied to disentangle the human dimension of fisheries bycatch and integrate locally-relevant criteria, such as set height or mesh strength of nets, that embrace the conceptual complexity of marine megafauna conservation research [79]. After risk baselines have been developed [94, 95], it is possible to compare feasible management and policy interventions. Finally, GIS-based scenarios that capture inter-annual variability and modifications of fishing gears [52] could be incorporated into ByRA to examine how the location and timing of risk is likely to change in the future, and anticipate at-risk areas in need of further monitoring and evaluation.

### 4.3. Conclusion

We created a spatially explicit management tool (ByRA) to better understand and characterize risk of bycatch posed by common fishing gears in data-limited small-scale fisheries. Three unique field sites, where substantial marine mammal bycatch has been reported, were systematically screened using existing data and a powerful form of visualization to map areas and seasons of concern in a region where distinct spatio-temporal patterns of bycatch risk had not been identified. ByRA employed a range of geospatial and participatory engagement techniques–including specific methods tailored for data scarce areas–to compile and analyze existing information about small-scale fisheries and better plan further research, bycatch mitigation, and species recovery and protection. This information enables managers to establish baselines, deliberate with stakeholders on the next steps for data acquisition, and identify interventions that are likely to mitigate bycatch risk in small-scale fisheries. It may also help these fisheries comply with European Commission and U.S. regulations [84, 96] that require efforts to reduce the acute threat of marine mammal bycatch to sustainable levels.

## Supporting information

S1 Data (DOCX)
