# Supplementary material for: Using GIS and stakeholder involvement to innovate marine mammal bycatch risk assessment in data-limited fisheries
Source: PLoS One. 2020 Aug 20;15(8):e0237835. doi: 10.1371/journal.pone.0237835 (PMC7446845; doi:10.1371/journal.pone.0237835)
Supplement: S1 Data — (DOCX) [file pone.0237835.s001.docx]

**PLoS One Supporting Information Appendix**

Article title: Using GIS and stakeholder involvement to innovate marine mammal bycatch risk assessment in data-limited fisheries

Authors: Verutes, G.M., Johnson, A.F., Caillat, M., Ponnampalam, L.S., Peter, C., Vu, L., Junchompoo, C., Lewison, R.L., Hines, E.M.

The following Supporting Information is available for this article:

**1. Methodology and Scoring Scheme**

*1.1 Methods and tool for risk assessment*

A Bycatch Risk Assessment (ByRA) was applied to estimate risk of bycatch based on the spatial and temporal coincidence of ranked probabilities of overlap between species and fishing occurrences. This methodology was adapted from previous species risk assessment approaches and tools [1,2] and expands on the productivity-susceptibility analyses developed specifically for important fish species in the United States and Australia [3,4]. By combining distribution maps of species and fishing activities, and rating each species-gear interaction in space and time, ByRA produced a series of GIS layers, showing risk scores for each site or region, and a map layer for each focal species classified by the relative amount of risk (high/medium/low) in three Southeast Asian field sites (SBTI, KUCG, and KGBR).

Risk of bycatch was calculated as *exposure*, or the degree a species experiences stress due to gear-specific threat (spatial/temporal overlap, threat intensity, likelihood of interaction, catchability, and current status of management strategies) and its *consequence*, defined as the resilience of a species to a stressor (age of maturity, reproductive strategy, population connectivity, local status of species) and its sensitivity (mortality and life stages affected by gear). Using information from primary and secondary sources along with an extensive review of the literature, we scored the interactions between two species (dugongs and Irrawaddy dolphins) and up to five categories of fishing gears present at each site. For some ratings, based on their ecological relevance and available information to score a given species-gear interaction, we either omitted or weighted (up/down) their importance in the average scores for exposure and consequence criteria. Finally, a Euclidean (straight-line) distance function from the origin (minimum score) to the average exposure and consequence scores was applied to estimate individual and cumulative bycatch risk on a cell-by-cell basis.

*1.2 Spatial data on species for the spatial overlap criterion*

To predict habitat suitability in two of three field sites, the modeling software Maxent (version 3.4.1) was used [5]. Maxent has been widely applied to build Ecological Niche Models (ENMs) and Species Distribution Models (SDMs). We used presence only data to quantify the statistical relationship between predictor environmental covariates at locations where a species had been observed versus ‘background’ locations in which no species had been observed within the study region. By doing so, we could identify habitat suitability levels within the study region; specifically, to calculate the relative occurrence rate (ROR) of dugongs (*Dugong dugon*) and Irrawaddy dolphins (*Orcaella brevirostris*) and estimate suitable habitat. Ultimately, Maxent outputs served as inputs to ByRA, specifically to calculate two exposure criteria: spatial overlap (between the distributions of species and gear) and encounter rates (likelihood of interaction between species and gear).

To identify the best parameters within Maxent and avoid overfitting, we used the R tool package ENMeval, designed for data partitioning and model execution [6,7]. ENMeval automatically executes Maxent several times to aid in identifying settings that balance model fit and predictive ability. For each Malaysian site, Maxent runs with different parameters and combinations of environmental data were tested to fit each model and evaluate their performance [8]. With each model, an AICc value, and different measurements related to the Area Under the Curve (AUC) were calculated [7,9].

We applied ENMeval with different sets of environmental variables. For each variable, regularization factors ranging from 0.5 to 4 (with a step of 0.5) were tested in ENMeval. A model with the lowest delta AICc value was considered the best performing model. After the appropriate parameters were identified by comparing AICc and AUC values for the test data [9], those parameters were used to run a cross-validation model in Maxent. We used the *k*-fold cross-validation option with 10-folds [7]. The method estimates errors around fitted functions and predictive performance on held-out data [10]. A jackknife test was conducted for each selected model to identify the importance of the different environmental variables within the model. Here, each of *n* occurrence localities was used for testing once. If after analyzing ENMeval outputs it was not possible to identify one best set of parameters, all the best models identified were run in Maxent and the resulting outputs compared.

*Model selection and outputs.* To identify the best model, we analyzed the different Maxent outputs. There is a lack of consensus regarding which validation statistic(s) are appropriate to identify the model that best predicts the data [7] and how to determine the appropriate threshold value which will divide the study area into suitable and not suitable habitat [11,12,13]. Given our goal to identify different level of habitat suitability within the study area and map marine mammal interaction with fishing activity within these areas, we computed three different evaluation metrics, two of which quantified model overfitting (OR_MTP_ and OR_10_), and also a visual inspection of the Maxent output maps:

1. Test AUC values (AUC_TEST_) - the area under the curve of the receiver operating characteristic plot based on the test data provides an indication of the model’s goodness of fit. Higher values indicate a better ability to discriminate between conditions at withheld versus background data.
2. Minimum training presence omission rate (OR_MTP_) - the proportion of test (withheld) data with Maxent output values lower than that corresponding to the training data with the lowest ranking value (referred to as the 0% training omission). Lower omission rates indicate less overfitting of the model.
3. 10% training omission rate (OR_10_) - the omission rate of test data, excluding the 10% of training data with the lowest predicted suitability (referred to as 10% training omission).
4. Visual inspection of Maxent output maps with the threshold between suitable and not suitable habitat corresponding to a 0% and 10% omission rate.

For the first three metrics, a t-test was run between models to detect if the difference observed across the 10 *k*-fold of those values was significant or not. If there was no significant difference, visual inspection of the Maxent output maps were conducted to select the appropriate model.

Maxent outputs included: 1) a curve illustrating the relationship between omission rates (proportion of occurrence present in not suitable habitat) and cumulative threshold, 2) a measure of the AUC validation statistics, 3) a table that summarizes the omission rate corresponding to a relative occurrence rate (ROR) threshold used to transform the probability map to a habitat suitability layer, 4) a prediction surface map, and 5) an analysis of the environmental variables relationship with these data and their overall contribution to the model.

*Three levels of habitat suitability.* To use Maxent outputs as species distribution inputs to the ByRA, we split continuous estimates of habitat suitability into classification levels. Once a threshold (either 0% or 10% OR) value was selected to transform probability into suitable versus unsuitable habitat, we produced maps of three levels of habitat suitability (low, medium and high). All grid cells with an ROR below the threshold value were classified as unsuitable habitat. The first category, *low suitability*, ranged from the omission rate threshold value to 50% of the maximum ROR of each species. The second category, *medium suitability*, ranged from 50% of the maximum ROR to 75% of the maximum ROR of each species. The final category, *high suitability*, ranged from 75% of the maximum ROR to the maximum ROR of each species.

*1.2.1 Dugongs*

In SBTI field site, the first aerial survey was conducted in 2010 over an eight-day period during the dry season. Then, from 2014 to 2016 additional surveys were commissioned over a 6-day period. Given the timing of dugong occurrence data available, it was not possible to split the data by seasonal weather (monsoonal) patterns, as in the KUCG field site. A total of 1360 dugong sightings were recorded with group sizes ranging from one to 43 individuals (S1 Table).

*Environmental covariates*. The environmental parameter, bathymetric slope, was calculated using a digital bathymetry layer created in GIS for the SBTI field site (as in Briscoe et al. [14]). Independent of the bathymetric slope variable being included, the Maxent models with the lowest AICc were always the ones with the LQHPT features and a regularization parameter of 3.5 (S2 Table). No significant difference (p > 0.05) was detected between these two models. A small difference in the medium level of habitat suitability could be observed, but this was unlikely to be significant (S1, S2, and S3 Fig). A corridor of highly suitable habitat was visible between Sibu-Tinggi Islands and the mainland (S1 Fig). The highest habitat suitability areas for dugongs were found mainly around the islands known to have extensive seagrass meadows. For both models, the environmental variables that contributed the most were distance to river mouths (40%) and then distance to land (35%). Both models correctly identified the test occurrence data as suitable habitat 88% of the time (S2 Table).

*1.2.2 Irrawaddy dolphins*

In KUCG, between one and 8 consecutive days of boat surveys were conducted before and after the monsoon season each year from 2008 to 2013. This regular survey interval offered the possibility to predict habitat usage of Irrawaddy dolphins by season, i.e., post-monsoon (March to May), at the heart of the dry season (June to August) and pre-monsoon (September to November) (S1 Table). A data merge of all seasons included 882 dolphin occurrences recorded in groups ranging from one to 45 individuals in size. While some dolphin sightings were recorded during the monsoon season (December to February), these data were not used because of the small sample size (*n=20*) and recurring challenges associated with difficult weather conditions and poor visibility, resulting in surveyors missing dolphins present in the area.

In KGBR, the boat surveys by Vu and colleagues [15] were the first ever conducted for the area. The team in Vietnam conducted three surveys over several days and months of the year 2014. In 2015, one survey over nine days was also completed. However, only two sightings of Irrawaddy dolphin were recorded over this two-year period. Maxent needs a minimum of five occurrences (Pearson et al. 2007). Habitat suitability for dolphins in KGBR was estimated using habitat preference parameters identified by Minton and colleagues (2011) and also in Thailand, the closest available data with a complete analysis of habitat suitability, by Jackson-Ricketts [16] (S3 Table, S4 Fig).

*Environmental covariates*. Environmental data known to influence the distribution of the Irrawaddy dolphins in their marine habitat such as sea surface temperature, pH, and salinity [15,17] were collected each time a dolphin was sighted in both the KUCG and KGBR sites. However, no environmental data outside these sighting points were available with high enough resolution to be spatially interpolated and used as covariates in Maxent. Consequently, only fixed physical environmental variables that could be mapped in a GIS were used; specifically, distance to river mouth, distance to land, and ocean depth (S1 Table). For depth information, we lacked a detailed bathymetric chart for Kuching Bay. This made it necessary to interpolate depth data collected by a boat-based fish finder during cetacean surveys. The acquired depth values followed boat transect lines and values between these lines were interpolated. The resulting bathymetry layer had linear artifacts for depth values and we chose not to use bathymetric slope as an environmental covariate in Maxent. Only one set of environmental parameters (not including slope) were tested for KUCG.  Maxent runs were evaluated with all occurrence data together (“All_Data_”) and with these occurrence data split by season (S4 Table).

*All sightings data.* When all dolphin occurrences were pooled together, two models had the same AIC scores, one with LQHP features and one with LQHPT features, and both using a regularization parameter of 1.0. The model (“All_Data_1”) with LQHPT features had a significantly (p < 0.05) better AUC of 90.58%, but the 10% and 0% omission rates were not significantly different (S4 Table). The environmental variable contribution rates to the model were also different between these two models. For the LQHP model, distance to land was the variable that contributed most to the model, whereas for the LQHPT model, both distance to river mouths and distance to land contributed the same amount (no significant difference of the contribution rate between these two variable based on t-test; p > 0.05).

Visual observation of the Maxent output maps showed some important differences (S5 Fig). Generally, both models predicted the most suitable habitat to be within the rivers and near river mouths. However, the medium habitat suitability range differed substantially, as this intermediate suitability level covered a smaller area in the LQHPT model. In addition, the edges of low habitat suitability areas extended further offshore in LQHP as compared to the LQHPT model.

*Sightings data by season.* When Irrawaddy dolphin occurrence data were split by season in KUCG, several models with different combination of Maxent features and regularization values had similar AICc values. For each of three seasons, two models with similar AICc (a difference of less than two points) were identified by the ENMeval function (S6, S7, S8, S9 and S10 Fig). The model with the lowest delta AICc value was considered the best model. For the post-monsoon and dry season, the models with the Threshold feature (“PoM_2” and “Dry_2”, respectively) had significantly higher mean AUC values (p < 0.05) than models without this feature class. For the dry season and pre-monsoon in KUCG, we selected models where distance to river mouths had the highest variable importance (55% and 49%, respectively) and there was strong discrimination on held out data (cross-validated AUCs of 94.02 and 87.48).

In terms of model overfitting, no significant differences were detected between the 10% training presence omission rates (S4 Table). Finally, a visual inspection of predicted suitability levels served to identify which habitat suitability map would be used as input to the ByRA. Overall, Maxent models that included the threshold features had a more patchy distribution of the medium level of habitat suitability and linear artifacts at the edge of the medium suitability level, which was a consequence of the depth covariate layer.

Across seasons in KUCG, the environmental covariates we tested did not contribute in the same way to model predictions. For the dry and pre-monsoon season, distance to river mouths provided the strongest contribution. During the post-monsoon season, however, it was the distance to land variable that contributed the most (S4 Table). The distribution of three habitat suitability levels also differed substantially across the three seasons. The entire spatial extent of suitable dolphin habitat went furthest offshore prior to the monsoon season and then moved closer to land during the dry season. The extent of highest habitat suitability level followed the same pattern; that is, it was centered in the river network or just outside major river mouths during the dry season and then extended further offshore before and after the monsoon (S6 Fig).

*1.3 Rankings for resilience criteria*

Resilience attributes were used in ByRA to describe the consequences (impact) to species by evaluating how a species population will respond and recover from impact. The literature on resiliency of dugongs and Irrawaddy dolphins was reviewed to compile rating scores for the consequence criteria related to age of maturity, reproductive strategy, population connectivity, and local status of species (S5 Table).

*1.3.1 Dugongs*

*Age of maturity.* Given their inconspicuous behavior, information about the life cycle of dugongs was sparse. According to research in Australia, the age of maturity of dugongs varies in space and time, between sexes, populations, and environmental conditions [18,19]. For females, a minimum age of maturity of six years was observed at Mabuiag Island [19]. In Marnington Island, the age of maturity ranged from 14.5 to 17.5 years for females and 15 to 16 years for males [18]. In Mabuiag Island, it was 6 to 7 years for females and 4 to 13 years for males [19]. Literature suggests an age of maturity greater than four years for both male and female dugongs.

*Reproductive strategy.* Calving interval is typically estimated using the length of gestation period and ratio of the number of mature females to number of pregnant females. Similar to age of sexual maturity, the calving interval of dugongs varies considerably [19] and is relatively long [20]. Based on observations of different populations, we estimated a calving interval between 3 and 7 years [18,19,21]. Maternal investment – time and energy spent beginning at conception [22] – is also high. Female dugongs can simultaneously be pregnant and nurse a calf [21]. While the gestation period of dugongs is relatively well known, information about the length of lactation is sparse.

*Population connectivity.* Dugongs have been observed to undertake micro-scale commuting between seagrass beds (<15km) and large-scale (>15km) movements. Some individuals undertook movements ranging from several hundreds of kilometers up to 1000km, driven by warmer waters or available seagrass meadows [23,24]. Large-scale movement, in which individuals did not stop at a feeding area, could be driven by spatial memory of foraging path or by socials cues [23]. Furthermore, dugongs can migrate long distances in response to habitat degradation [25].

*Local status of species.* At the international level, dugongs have been categorized as “vulnerable” by the IUCN Red List [26]. In Peninsular Malaysia, the Fisheries Act 2985 and the Fisheries Regulations 1999 (Control of Endangered Species of Fish) aim to protect dugongs [14,27]. The Department of Fisheries Malaysia implemented a National Plan of Action for Dugongs in 2011 to protect, conserve and manage dugongs and their habitats [27,28].

*1.3.2 Irrawaddy dolphins*

The literature on resiliency of Irrawaddy dolphins when recovering from a threat, particularly in Malaysia and Vietnam, was limited. To rank resilience criteria for this species, we reviewed studies from Trat Bay, Thailand [16,29,30], which neighbors Cambodia to the north along the Gulf of Thailand. A genetic diversity study by Caballero and colleagues [31] suggests connectivity between populations in India, Thailand and Cambodia, but less is known about dolphins using the coastal waters of the KGBR and KUCG sites. Regional studies of Irrawaddy suggest that adult length is achieved between 4 and 6 years old [32] and age at reproductive maturity for both females and males is between 3 to 6 years [33]. Irrawaddy dolphins are not fully weaned until 2 years of age, with an average weaning age of about 24 months. From birth to about six months, calf nutrition comes entirely from the mother, who invests heavily in her young [33]. The [IUCN](https://en.wikipedia.org/wiki/IUCN) classifies this species as [“endangered](https://en.wikipedia.org/wiki/Endangered)” or a decreasing population trend [34] due in large part to incidental capture in fishing nets.

*1.4 Fisheries gear data for estimating the spatial overlap criteria and literature review for estimating scores for remaining exposure and consequence (sensitivity) criteria*

Existing aerial surveys in SBTI and boat surveys in KUCG were used to map the distribution and intensity of fishing gear use [17,27]. The MareCet Research Organization also conducted in-depth interviews of fishers in SBTI during 2010 and 2016 as part of social science research for dugong conservation. Surveyors asked respondents to draw approximate fishing grounds around Sibu-Tinggi and neighboring islands. This qualitative information was later merged into the kernel density maps by gear type, assuming the lowest intensity score for gear-specific fishing areas not identified by aerial surveys (S11 Fig). In KGBR, the Vietnam Marine Megafauna Network interviewed fishers opportunistically in 2014 and again in 2016 to characterize fishing activities by gear type. However, it was not possible to combine these interview data with boat-based observations of fisheries in the KGBR study area because their data sheets noted only the number of flags displayed at the water surface (S1 Table). Flags attached to gears allow for a qualitative estimate of the distribution of fishing activities within an area but do not provide specifics about the gear itself. Consequently, this criterion (intensity of gear use) was omitted as an exposure attribute in KGBR (S6 Table).

The peer-review and grey literature on exposure and consequence-sensitivity of dugongs and Irrawaddy dolphins to five categories of fishing gears was reviewed to compile ratings for the remaining risk criteria (S5 and S6 Table) that characterize extent to which a population was exposed to a stressor. Specifically, these criteria described the overlap between a species’ distribution and the extent of fishing activity in space and time. Also accounted for was the relative likelihood of capture by gear “catchability” and the degree to which current management had been identified and/or implemented with respect to the fishing gear in question. Lastly, we scored two criteria related to the consequence-sensitivity of a species population – i.e., how it will respond and recover from the impact (mortality/severity and life stages affected by gear).

*1.4.1 Exposure*

*Temporal overlap.* Irrawaddy dolphins use the coastal, estuarine and riverine waters of Kuching Bay [35]. Scientific surveys conducted in KUCG along with interviews of fishers indicated that dolphins are generally present year-round (C. Peter, personal communication, August 4, 2017). Nets were reported to be used all year within these areas (Table 27 in [36]). Less is known about Irrawaddy dolphins in the waters around KGBR and Cambodia.

*Likelihood of capture.* The scientific community has identified gillnets as a serious threat to dugongs and dolphins but this has been largely unquantified [37,38,39]. There were limited reports in Southeast Asia of dugong bycatch from nets due to a long tradition of hunting the animal for their body parts and meat, and consequently the challenges associated with identifying cause of death. The research by Jaaman et al. [40] in East Malaysia suggested that bycatch rates, particularly in gillnets, may be unsustainably high. Even if a dugong encounters a net with weak mesh, the animal may scare and roll around the net or ropes after impact (A. Ilankgakoon, personal communication, March 8, 2018). For dugongs, incidental catch in push net trawlers has been reported [41]. In Thailand, dolphins were observed following trawlers and sometimes swam too close to the nets, causing mass drownings with multiple dolphins found stranded together (W. Laovechprasit, personal communication, August 4, 2017). While the marine megafauna monitoring network in Trat Bay, Thailand found part of a trap around a dolphin [29], there were no reports of pots and traps entangling dolphins in KUCG.

*Current status of management.* Dugongs are among the species protected under the Malaysia Fisheries Act [42] and Fisheries Regulations. The Malaysian government committed to protecting 10% of the marine environment by 2020 [27]. The Sultan Iskandar Marine Park, inside the SBTI field site, extends 2nm from low tide mark of the islands (S12 Fig). The marine park, gazetted in 1994, is a no-take zone for fishing. Most of the bycatch threats to dugongs and other megafauna occur in the areas beyond the boundaries of the park. Additional areas around Sibu-Tinggi Islands (cross-hatching in S12 Fig) have been proposed for fisheries management [27]. The current status of management in KUCG and KGBR was mapped in GIS based on identified conservation areas (S12 Fig) [15,17,35].

*1.4.2 Consequence-sensitivity*
 *Mortality.* The diving time of dugongs is usually less than 12 minutes [23,43]. If the animal is entangled by fishing gear and not retrieved within their lung capacity, it will drown. The scientific community considers the severity of dugongs caught in nets to be lethal and interactions with nets are known to be a significant cause of dugong mortality [44,45]. To our knowledge, there have been no studies conducted to quantify the severity of encounters between dugongs and other types of fishing gears (trawls, hook and line, etc.). Based on the biology and behavior of dugongs and how different gears function in the water, we estimated the impact from hook and line as negligible, while trawls and pots and traps to be sublethal [14,46]. Similar severity ratings were used for Irrawaddy dolphin, except trawls were scored as lethal instead of sublethal [29,30].

*Life stages affected by gear.*  Along the Mersing Archipelago, many dugongs have been lost in the past three years as a result of bycatch in and around the SBTI field site. They were mainly juveniles and young adults. For each dugong with reproductive potential lost in SBTI, it is estimated to take 15 years to replace another one in the population (L. Ponnampalam, personal communication, August 4, 2017). For Irrawaddy dolphins (KUCG and KGBR sites), we zeroed out the rating score for this attribute due to lack of information and ecological relevance.

*1.5 Limitations and simplifications*

Despite an extensive literature review, it was not possible to justify exposure and consequence criteria ratings for all interactions between two species and five fishing gears in three field sites of Southeast Asia. Two expert working groups were organized, one in California (July 2017) and the other in Thailand (August 2017) to systematically review criteria descriptions and reach a consensus on ratings when references were not available. This convening of partners served to define the scoring bins (1-3) for each criterion and parameterize ByRA’s variable weighting structure for data quality and criteria importance scores (S5, S6 and S7 Table).

Maxent enables users to tune numerous settings for creating complex models with many parameters [10]. However, it is subject to several challenges related to model specifications [7,47] and interpretation of outputs [8,13]. One issue is the lack of controls for over-parameterization and sampling bias. Here, the choice of the best available model depended on the model’s ability to predict independent test data only, and not the model complexity. Consequently, Maxent is known to overfit the data [7,9,13] . Given the paucity of available environmental data for the SBTI and KUCG field sites, we constrained our environmental predictors to a maximum of four variables (S1 Table). It is possible that some of the selected models were overfit based on a limited set of environmental variables for a potentially larger number of Maxent parameters.

**Supplementary References**

1. Samhouri JF, Levin PS. Linking land-and sea-based activities to risk in coastal ecosystems. Biological Conservation. 2012 Jan 1;145(1):118-29.
2. Arkema KK, Verutes G, Bernhardt JR, Clarke C, Rosado S, Canto M, Wood SA, Ruckelshaus M, Rosenthal A, McField M, De Zegher J. Assessing habitat risk from human activities to inform coastal and marine spatial planning: a demonstration in Belize. Environmental Research Letters. 2014 Nov 18;9(11):114016.
3. Patrick WS, Spencer P, Link J, Cope J, Field J, Kobayashi D, Lawson P, Gedamke T, Cortés E, Ormseth O, Bigelow K. Using productivity and susceptibility indices to assess the vulnerability of United States fish stocks to overfishing. Fishery Bulletin. 2010;108(3):305-22.
4. Hobday AJ, Smith AD, Stobutzki IC, Bulman C, Daley R, Dambacher JM, Deng RA, Dowdney J, Fuller M, Furlani D, Griffiths SP. Ecological risk assessment for the effects of fishing. Fisheries Research. 2011 Mar 1;108(2-3):372-84.
5. Phillips SJ, Dudík M, Schapire RE. Maxent software for modeling species niches and distributions (Version 3.4.1). Available from: <http://biodiversityinformatics.amnh.org/open_source/maxent/>.
6. R Core Team. R: A language and environment for statistical computing. 2017.
7. Muscarella R, Galante PJ, Soley‐Guardia M, Boria RA, Kass JM, Uriarte M, Anderson RP. ENM eval: An R package for conducting spatially independent evaluations and estimating optimal model complexity for Maxent ecological niche models. Methods in Ecology and Evolution. 2014 Nov;5(11):1198-205.
8. Rhoden CM, Peterman WE, Taylor CA. Maxent-directed field surveys identify new populations of narrowly endemic habitat specialists. PeerJ. 2017 Jul 31;5:e3632.
9. Warren DL, Glor RE, Turelli M. ENMTools: a toolbox for comparative studies of environmental niche models. Ecography. 2010 Jun;33(3):607-11.
10. Elith J, Phillips SJ, Hastie T, Dudík M, Chee YE, Yates CJ. A statistical explanation of Maxent for ecologists. Diversity and distributions. 2011 Jan 1;17(1):43-57.
11. Liu C, Berry PM, Dawson TP, Pearson RG. Selecting thresholds of occurrence in the prediction of species distributions. Ecography. 2005 Jun;28(3):385-93.
12. Pearson RG, Raxworthy CJ, Nakamura M, Townsend Peterson A. Predicting species distributions from small numbers of occurrence records: a test case using cryptic geckos in Madagascar. Journal of biogeography. 2007 Jan;34(1):102-17.
13. Warren DL, Seifert SN. Ecological niche modeling in Maxent: the importance of model complexity and the performance of model selection criteria. Ecological applications. 2011 Mar;21(2):335-42.
14. Briscoe DK, Hiatt S, Lewison R, Hines E. Modeling habitat and bycatch risk for dugongs in Sabah, Malaysia. Endangered Species Research. 2014 Jun 13;24(3):237-47.
15. Long V, Tho TA, Hung NN, Duy L. Conservation of cetaceans in Kien Giang Biosphere Reserve, Vietnam. Conservation Leadership Program. 2017;3234115.
16. Jackson-Ricketts J. Diet, Life History, Habitat, and Conservation of Irrawaddy Dolphins (Orcaella brevirostris) in the Gulf of Thailand (Doctoral dissertation, UC Santa Cruz).
17. Peter C, Poh AN, Ngeian J, Tuen AA, Minton G. Identifying habitat characteristics and critical areas for Irrawaddy dolphin, Orcaella brevirostris: implications for conservation. In Naturalists, explorers and field scientists in South-East Asia and Australasia 2016 (pp. 225-238). Springer, Cham.
18. Marsh H, Heinsohn GE, Marsh LM. Breeding Cycle, Life History and Population Dynamics of the Dugong, Dugon dugon (Sirenia: Dugongidae. Australian Journal of Zoology. 1984;32(6):767-88.
19. Kwan D. Towards a sustainable indigenous fishery for dugongs in Torres Strait: A contribution of empirical data analysis and process (Doctoral dissertation, James Cook University).
20. Mann J. Parental behavior. In Encyclopedia of marine mammals 2009 Jan 1 (pp. 830-836). Academic Press.
21. Marsh H. The Life History, Pattern of Brcedhg, and population Dynamics of the Dugong. 1995.
22. Hayssen V. Empirical and theoretical constraints on the evolution of lactation. Journal of Dairy Science. 1993 Oct 1;76(10):3213-33.
23. Sheppard JK, Preen AR, Marsh H, Lawler IR, Whiting SD, Jones RE. Movement heterogeneity of dugongs, Dugong dugon (Müller), over large spatial scales. Journal of Experimental Marine Biology and Ecology. 2006 Jun 27;334(1):64-83.
24. Hobbs JP, Frisch AJ, Hender J, Gilligan JJ. Long-distance oceanic movement of a solitary dugong (Dugong dugon) to the Cocos (Keeling) Islands. Aquatic Mammals. 2007;33:175-8.
25. Preen A, Marsh H. Response of dugongs to large-scale loss of seagrass from Hervey Bay, Queensland Australia. Wildlife Research. 1995;22(4):507-19.
26. Marsh H, Sobtzick S. Dugong dugon. The IUCN Red List of Threatened Species 2015: e. T6909A43792211.
27. Ponnampalam LS, Izmal JF, Adulyanukosol K, Ooi JL, Reynolds JE. Aligning conservation and research priorities for proactive species and habitat management: the case of dugongs Dugong dugon in Johor, Malaysia. Oryx. 2015 Oct;49(4):743-9.
28. DOFM D. National Plan of Action for Dugongs 2011 Putrajaya, Malaysia. [In Bahasa Malaysia].
29. Junchompoo C, Monanunsap S, Penpein C. Population and Conservation Status of Iirawaddy Dolphins (Orcaella brevirostris) in Trat Bay, Trat Province, Thailand.
30. Hines E, Strindberg S, Junchompoo C, Ponnampalam LS, Ilangakoon AD, Jackson-Ricketts J, Mananunsap S. Line transect estimates of Irrawaddy dolphin abundance along the eastern Gulf Coast of Thailand. Frontiers in Marine Science. 2015 Sep 3;2:63.
31. Caballero S, Dove V, Jackson-Ricketts J, Junchompoo C, Cohen S, Hines E. Mitochondrial DNA diversity and population structure in the Irrawaddy dolphin (Orcaella brevirostris) from the Gulf of Thailand and the Mekong River. Marine Mammal Science. 2018;35(1):300-10.
32. Stacey, PJ, Arnold, PW. Orcaella brevirostris. Mammalian Species, 1999;616:1–8.
33. Stacey PJ, Leatherwood S. The Irrawaddy dolphin, Orcaella brevirostris: a summary of current knowledge and recommendations for conservation action. Asian Marine Biology. 1997 Dec 1;14:195-214.
34. Minton G, Smith BD, Braulik GT, Kreb D, Sutaria D, Reeves R. Orcaella brevirostris. The IUCN Red List of Threatened Species. 2017 Dec.
35. Minton G, Peter C, Tuen AA. Distribution of small cetaceans in the nearshore water of Sarawak, East Malaysia. Raffles Bulletin of Zoology. 2011 Feb 28;59(1).
36. DOFM D. Perangkaan Tahunan Perikanan 2011 (Annual Fisheries Statistics 2011).
37. Read AJ. The looming crisis: interactions between marine mammals and fisheries. Journal of Mammalogy. 2008 Jun 5;89(3):541-8.
38. Lewison RL, Soykan CU, Franklin J. Mapping the bycatch seascape: multispecies and multi‐scale spatial patterns of fisheries bycatch. Ecological Applications. 2009 Jun;19(4):920-30.
39. Reeves RR, McClellan K, Werner TB. Marine mammal bycatch in gillnet and other entangling net fisheries, 1990 to 2011. Endangered Species Research. 2013 Mar 21;20(1):71-97.
40. Jaaman SA, Lah-Anyi YU, Pierce GJ. The magnitude and sustainability of marine mammal by-catch in fisheries in East Malaysia. Journal of the Marine Biological Association of the United Kingdom. 2009 Aug;89(5):907-20.
41. Hines E, Adulyanukosol K, Duffus D, Dearden P. Community perspectives and conservation needs for dugongs (Dugong dugon) along the Andaman coast of Thailand. Environmental Management. 2005 Nov 1;36(5):654-64.
42. DOFM D. Fisheries Act of 1985. Available at: http://www.dof.gov.my/dof2/resources/user_1/UploadFile/AKTA_PERATURAN/Akta_A1413(BM)_-_Akta_Perikanan_(Pindaan)_2012.pdf. Accessed 2018 November 21.
43. Chilvers BL, Delean S, Gales NJ, Holley DK, Lawler IR, Marsh H, Preen AR. Diving behaviour of dugongs, Dugong dugon. Journal of Experimental Marine Biology and Ecology. 2004 Jun 30;304(2):203-24.
44. Marsh H. Evaluating management initiatives aimed at reducing the mortality of dugongs in gill and mesh nets in the Great Barrier Reef World Heritage Area. Marine Mammal Science. 2000 Jul;16(3):684-94.
45. Dolar ML, Jefferson TA, Marsh H, Wang JY, Estacion J. Report of the Second Workshop on The Biology and Conservation of Small Cetaceans and Dugongs of South East Asia. Perrin WF, Reeves RR, editors. UNEP-CMS; 2005.
46. Adulyanukosol K, Poovachiranon S. Dugong (Dugong dugon) and seagrass in Thailand: present status and future challenges.
47. Phillips SJ, Dudík M. Modeling of species distributions with Maxent: new extensions and a comprehensive evaluation. Ecography. 2008 Apr;31(2):161-75.

**Supplementary Tables**

**S1 Table. Summary of spatial data used to map the distribution of marine mammals and fishing activities and for each field site.**

|  |  | **SBTI** | **KUCG** | **KGBR** |
| --- | --- | --- | --- | --- |
|  | **References  for survey methodology** | Ponnampalam  et al. 2015 | Peter  et al. 2016 | Vu  et al. 2017 |
|  | **Years** | 2010, 2014-2016 | 2008-2013 | 2014-2015 |
|  | **Focal species** | *Dugong  dugon* | *Orcaella*  *brevirostris* | *Orcaella brevirostris* |
| **Animal  sightings** | **Post-monsoon** |  | 364 |  |
|  | **Dry season** | 1360 | 348 |  |
|  | **Pre-monsoon** |  | 150 |  |
|  | **Monsoon** |  | 20 |  |
|  | **Total** | 1360 | 882 | 2 |
| **Environ- mental variables** | **Depth** | x | x | x |
|  | **Distance to land** | x | x | x |
|  | **Distance to  river mouth** | x | x | x |
|  | **Bathymetric slope** | x |  |  |
| **Vessel sightings by fishery** | **Pots and traps** | 33 | 133 | - |
|  | **Hook and line** | 311 | 230 | - |
|  | **Trawls** | 115 | 11 | - |
|  | **Nets** | 2152 | 1086 | - |
|  | **Longlines** | 0 | 0 | - |
|  | **Total** | 2611 | 1460 | 56 |

**S2 Table . Summary of Maxent runs with the occurrence data for SBTI.** Outputs show: 1) the average contribution rate for each variable (and standard deviation). The variable with the largest effect is shown in bold; 2) the Maxent features: L=Linear, Q=Quadratic, H=Hinge, P=Product and T=Threshold; 3) the average AUC values from the test data (AUC_TEST_) with standard deviation, the average (and standard deviation) omission rate of the test data with the 10% threshold defined with the training data (OR_10_); 4) the average (and standard deviation) omission rate of the test data with the 0% threshold defined with the training data (OR_MTP_). Grey shading indicates the model selected.

|  |  | **SBTI_1** | **SBTI_2** |
| --- | --- | --- | --- |
| **Environmental variable contributions (%)** | Distance to river mouth | **40.10  (0.96)** | **40.45  (0.58)** |
|  | Distance to land | 34.96 (0.94) | 35.20 (1.26) |
|  | Depth | 24.78 (0.86) | 24.35 (1.12) |
|  | Slope | 16.34 (0.03) | - |
| **Maxent parameters** | Maxent features | LQHPT | LQHPT |
|  | Regularization factor | 3.5 | 3.5 |
| **Maxent validation statistics  (%)** | AUC_TEST_ | 88.48 (0.01) | 88.47 (0.01) |
|  | OR_10_ | 10.40 (0.04) | 10.32 (0.04) |
|  | R_MTP_ | 0.08 (0.002) | 0.08 (0.0024) |

**S3 Table. Rule-based GIS approach to map Irrawaddy dolphin habitat suitability in KGBR.**

| **Criteria** | **Rule** | **Low suitability** | **High suitability** |
| --- | --- | --- | --- |
| 1. **Depth** | 0 to 15m | must be | must be |
| 1. **Proximity to  major river mouths** | Less than 25km | can be (either 2 or 3) | must be |
| 1. **Proximity to land** | Less than 10km | can be (either 2 or 3) | must be |

**S4 Table. Summary of Maxent runs with the occurrence data for KUCG field site pooled together (“All_Data_” models) or divided by season (PoM, Dry, PrM).** Outputs show: 1) the average contribution rate for each variable (and standard deviation). The variable with the largest effect is shown in bold; 2) the Maxent features: L=Linear, Q=Quadratic, H=Hinge, P=Product and T=Threshold; 3) the average AUC values from the test data (AUC_TEST_) with standard deviation, the average (and standard deviation) omission rate of the test data with the 10% threshold defined with the training data (OR_10_); 4) the average (and standard deviation) omission rate of the test data with the 0% threshold defined with the training data (OR_MTP_). Asterisks indicate a significant difference as compared to other models that used the same environmental covariates*.* Grey shading indicates the model selected for each scenario.

|  |  | **All_ Data_1** | **All_ Data_2** | **PoM _1** | **PoM _2** | **Dry _1** | **Dry _2** | **PrM _1** | **PrM _2** |
| --- | --- | --- | --- | --- | --- | --- | --- | --- | --- |
| **Environmental variable contrib-utions (%)** | Distance to river mouth | 35.45 (0.49) | **39.46 (1.32)** | 31.71 (1.88) | 26.87 (1.15) | **55.26 (2.18)** | **49.98 (1.57)** | **48.68 (2.94)** | **45.23 (2.73)** |
|  | Distance  to land | **41.57 (0.58)** | 39.10 (1.03) | **47.42 (2.00)** | **49.87 (1.19)** | 22.13 (1.93) | 25.50 (1.29) | 23.82 (2.11) | 28.27 (2.52) |
|  | Depth | 22.97 (0.75) | 21.43 (0.48) | 20.86 (0.98) | 23.25 (1.17) | 22.61 (0.68) | 24.52 (0.81) | 27.94 (1.51) | 26.50 (1.62) |
| **Maxent param-eters** | Maxent features | LQHPT | LQHP | LQHP | LQHPT | LQHP | LQHPT | LQHP | LQHPT |
|  | Regular-ization factor | 1 | 1 | 1.5 | 1.5 | 1.5 | 1.5 | 3.5 | 3.5 |
| **Maxent valida-tion statis-tics  (%)** | AUC_TEST_ | 90.58^*^ (0.01) | 87.91 (0.02) | 88.54 (0.03) | 91.43^*^ (0.02) | 94.02 (0.01) | 95.68^*^ (0.01) | 87.48 (0.04) | 87.55 (0.04) |
|  | OR_10_ | 11.48 (0.03) | 10.16 | 10.0 (0.06) | 11.21 (0.05) | 9.94 (0.07) | 11.13 (0.06) | 10.0 (0.10) | 10.0 (0.10) |
|  | OR_MTP_ | 0.13 (0.004) | 0.13 (0.004) | 0.3 (0.01) | 0.3 (0.01) | 0.0 (0.00) | 0.0 (0.00) | 0.0 (0.00) | 0.0 (0.00) |

**S5 Table. Consequence criteria scores for five fishing gears categories across three field sites.** Showing scores contributing to highest risk (3), intermediate risk (2) and lowest risk (1). Scores of 0 were applied to irrelevant criteria or if not enough data exists and, consequently, these criteria were omitted from the bycatch risk equation. ‘N/A’ indicates gears not present in the field site. Data quality (DQ) and variable weights (W) applied as a weighted average for a site and specific gear-species interaction are listed in the Notes column.

| ***Consequence-Sensitivity*** | | **SBTI** (dugongs) | **KUCG** (dolphins) | **KGBR** (dolphins) | **Notes** |
| --- | --- | --- | --- | --- | --- |
| **Mortality** | | | | |  |
|  | nets | 3 | 3 | 3 |  |
|  | trawls | 2 | 3 | 3 | SBTI DQ = 3 |
|  | pots and traps | 2 | 2 | 2 |  |
|  | longlines | N/A | N/A | 1 |  |
|  | hook and line | 1 | 1 | 1 |  |
| **Life stages affect by gear** | | |  |  |  |
|  | nets | 0 | 2 | 0 |  |
|  | trawls | 2 | 0 | 0 |  |
|  | pots and traps | 0 | 0 | 0 |  |
|  | longlines | N/A | N/A | 0 |  |
|  | hook and line | 0 | 0 | 0 |  |

| ***Consequence-Resilience*** | **SBTI** (dugongs) | **KUCG** (dolphins) | **KGBR** (dolphins) | **Notes** |
| --- | --- | --- | --- | --- |
| **Age of maturity** | 3 | 3 | 3 |  |
| **Reproductive strategy** | 3 | 2 | 2 |  |
| **Population  connectivity** | 2 | 3 | 3 |  |
| **Local status of  the species** | 3 | 3 | 3 |  |

**S6 Table. Exposure criteria scores for five fishing gears across three field sites.** Showing scores contributing to highest risk (3), intermediate risk (2) and lowest risk (1). Scores of ‘SEC’ indicates spatially explicit criteria layers shown in S11, S12 and S13 Fig. Scores of ‘0’ were applied to irrelevant criteria or if not enough data exists and, consequently, these criteria were omitted from the bycatch risk equation. ‘N/A’ indicates gears not present in the field site. Data quality (DQ) and variable weights (W) applied as a weighted average for a site and specific gear-species interaction are listed in the Notes column.

| ***Exposure criteria*** | | **SBTI** (dugongs) | **KUCG** (dolphin) | **KGBR** (dolphins) | **Notes** |
| --- | --- | --- | --- | --- | --- |
| **Spatial overlap** | | SEC | SEC | SEC |  |
| **Temporal overlap** | | | | |  |
|  | nets | 2 | 3 | 3 | KUCG DQ = 1, KGBR DQ = 3 |
|  | trawls | 2 | 2 | 2 | KUCG DQ = 3 |
|  | pots and traps | 3 | 3 | 3 |  |
|  | longlines | N/A | N/A | 2 |  |
|  | hook and line | 2 | 3 | 3 | KUCG DQ = 3 |
| **Intensity of gear use** | |  |  |  | S11 Fig |
|  | nets | SEC | SEC | 0 |  |
|  | trawls | SEC | SEC | 0 |  |
|  | pots and traps | SEC | SEC | 0 |  |
|  | longlines | N/A | N/A | 0 |  |
|  | hook and line | SEC | SEC | 0 |  |
| **Likelihood of interaction between gear and species** | | SEC | SEC | SEC | SBTI W = 1, KUCG W = 1; S13 Fig |
| **Likelihood of capture by gear (“catchability”)** | |  |  |  | SBTI W = 1; KGBR W = 1 |
|  | nets | 3 | 3 | 3 |  |
|  | trawls | 2 | 2 | 2 |  |
|  | pots and traps | 1 | 1 | 1 | KUCG DQ = 3 |
|  | longlines | N/A | N/A | 1 | KGBR DQ = 3 |
|  | hook and line | 1 | 1 | 1 |  |
| **Current status  of management** | | SEC | SEC | SEC | S12 Fig |

**S7 Table. Data quality ratings for bycatch risk assessment.**

| **Data quality** | **Description** | **Example** |
| --- | --- | --- |
| **1** (green) | **Best data.** Substantial information is available to support the score and is based on data collected in the study region (or nearby) for the species in question. | **Animal sightings -** Distribution data collected during line transect survey. Data could be used to estimate relative abundance with robust methodologies and measurements of uncertainties. |
| **2** (yellow) | **Adequate data.** Information is based on data collected outside the study region, may be based on related species, may represent moderate or insignificant statistical relationships. | **Habitat suitability -** Estimated using non-modeled distribution methodology, minimal environmental variables collected. |
| **3** (red) | **Limited data.** No empirical literature exists to justify scoring for the species but a reasonable inference can be made. | **Fishing effort / gear type densities -** Sparse or incomplete data, no geospatial or precise localization of the fishing effort/gear distribution. |

**5. Supplementary Figures**


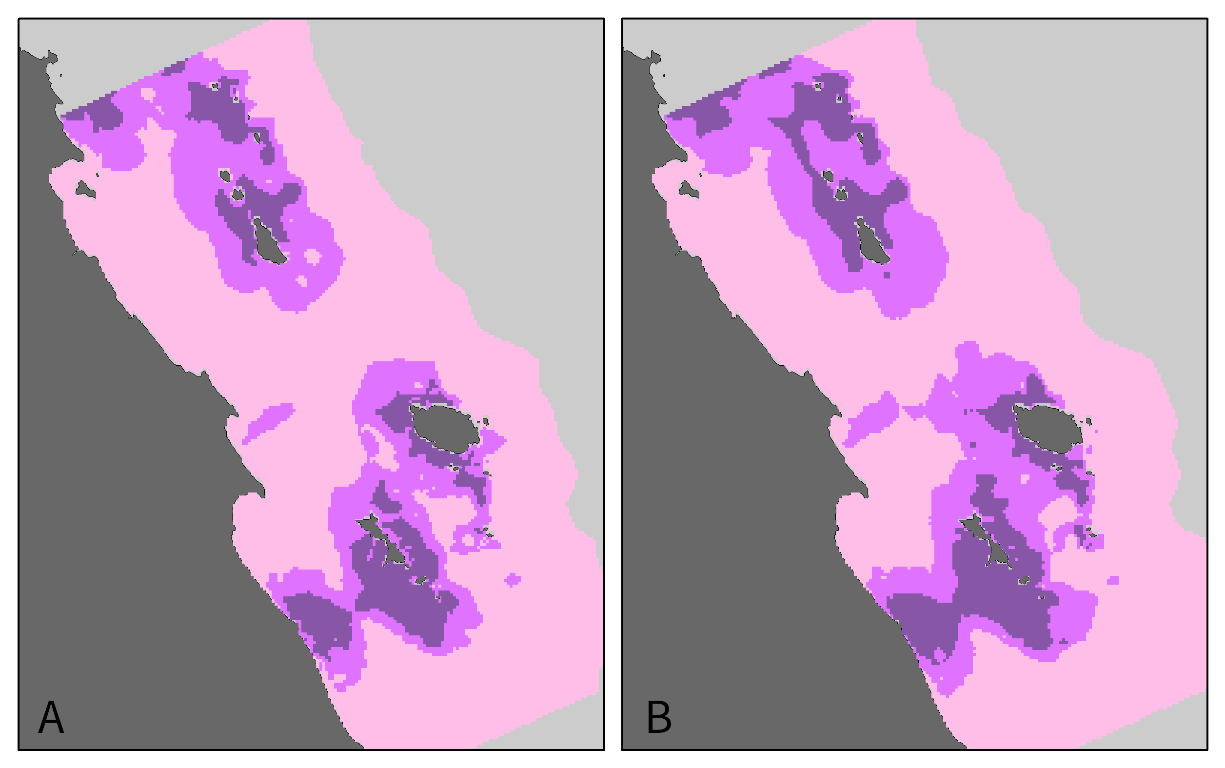

**S1 Fig. Habitat suitability model outputs for dugongs in SBTI.** (A) with and (B) without bathymetric slope included as an environmental covariate. Warmer colors indicate higher predicted suitability.


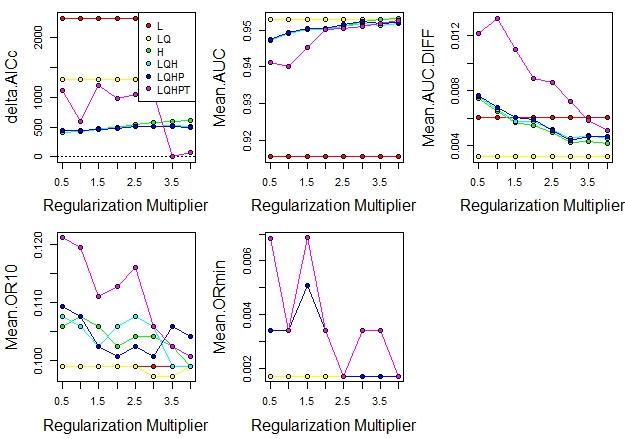


**S2 Fig. ENMeval outputs for SBTI with bathymetric slope covariate.** Model of dry season only occurrence data with the environmental covariates: distance to river mouths, distance to land, water depth, and bathymetric slope.


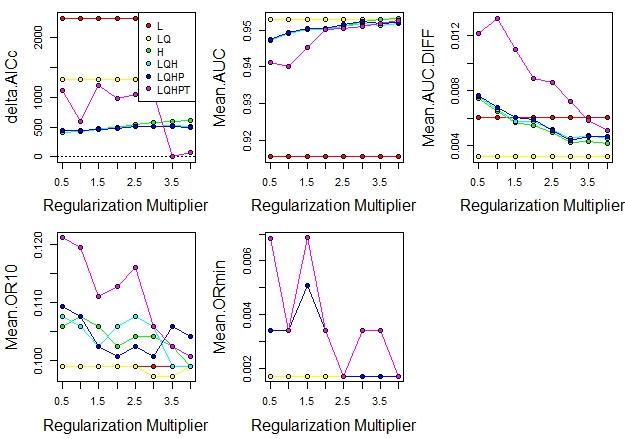


**S3 Fig.** **ENMeval outputs for SBTI without bathymetric slope covariate.** Model of dry season only occurrence data with the environmental covariates: distance to river mouths, distance to land, and water depth.

*
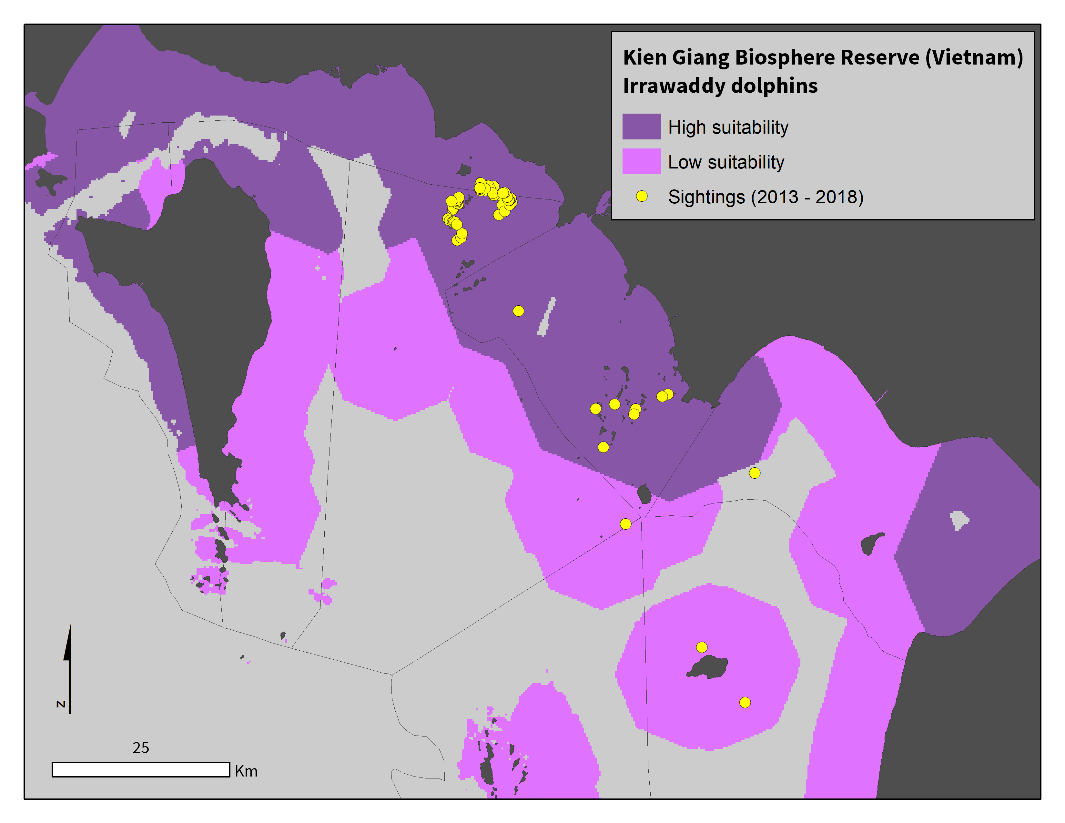
***S4 Fig.** **Rule-based GIS approach for estimating habitat suitability in KGBR.** Yellow dot-symbols show sightings locations of Irrawaddy dolphins (*Orcaella brevirostris*) acquired from in-country partners after the ByRA. Warmer colors indicate higher predicted suitability.


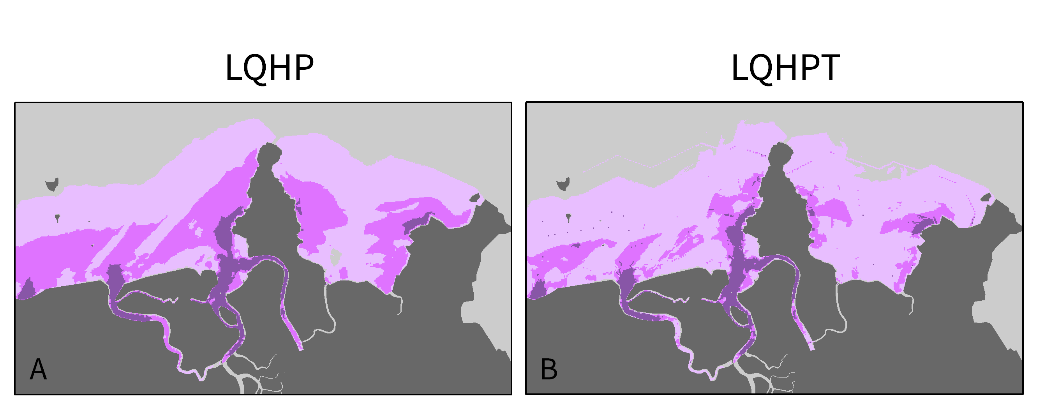


**S5 Fig.** **Habitat suitability model for Irrawaddy dolphins in KUCG with all the data pooled.** (A) LQHP and (B) LQHPT feature classes allowed. Warmer colors indicate higher predicted suitability.


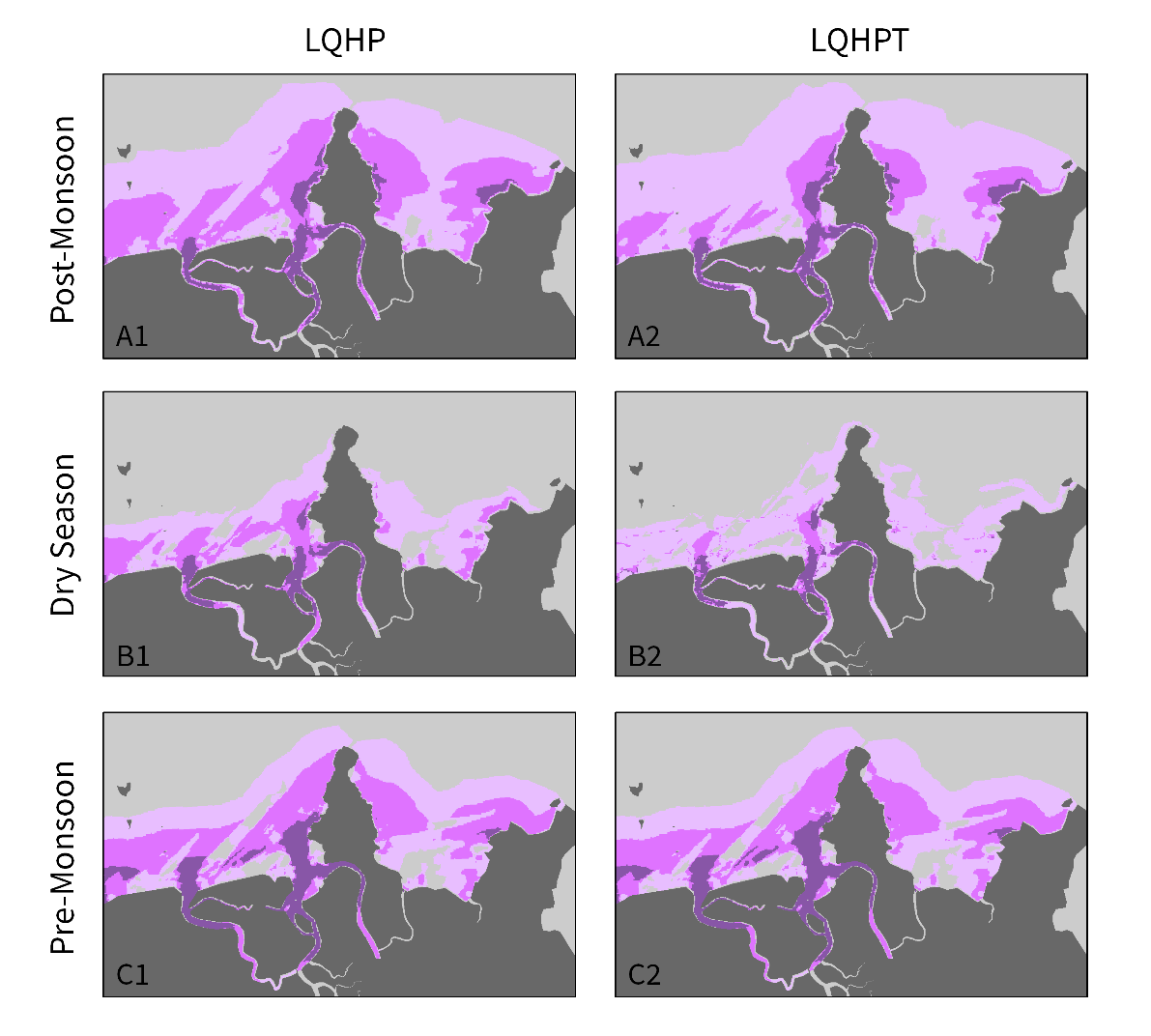


**S6 Fig.** **Habitat suitability model outputs for Irrawaddy dolphins in KUCG.** Panels organized by season (A-C) and Maxent feature classes (1-2). Warmer colors indicate higher predicted suitability.


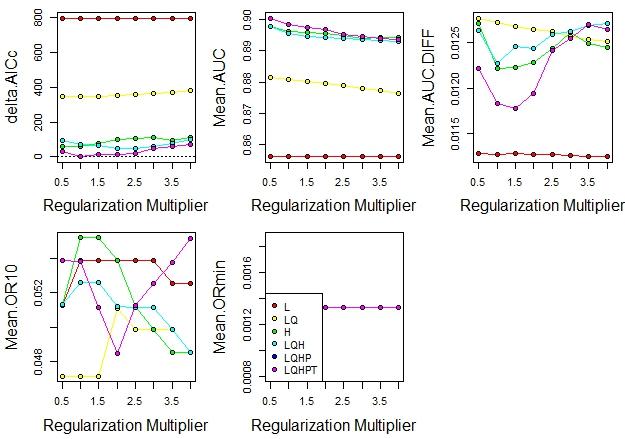


**S7 Fig.** **ENMeval outputs for KUCG with all data pooled.**  Model includes environmental covariates: distance to river mouths, distance to land and bathymetric depth.


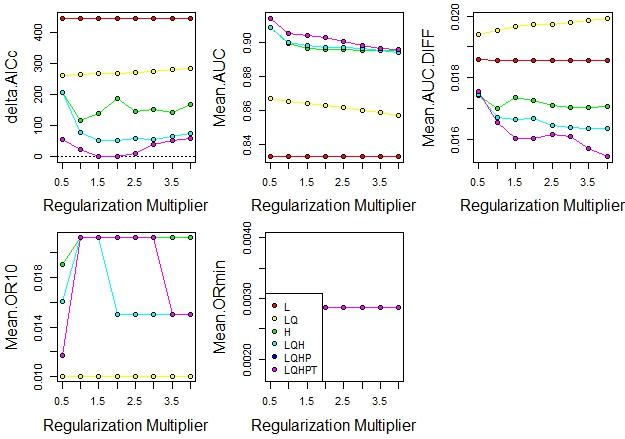


**S8 Fig.** **ENMeval outputs for KUCG with the post-monsoon occurrence data.**  Model includes environmental covariates: distance to river mouths, distance to land and bathymetric depth.


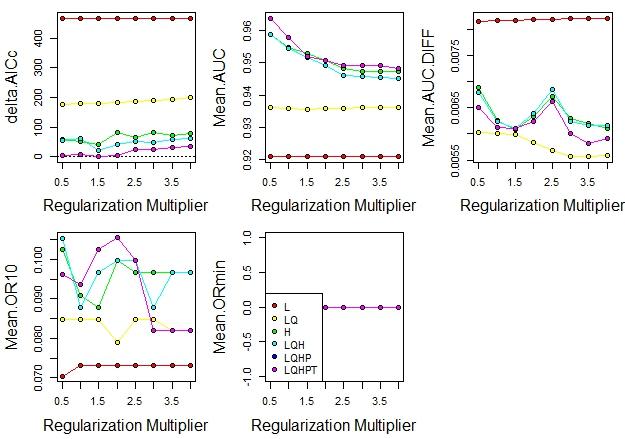


**S9 Fig.** **ENMeval outputs for KUCG with the dry season occurrence data.**  Model includes environmental covariates: distance to river mouths, distance to land and bathymetric depth.


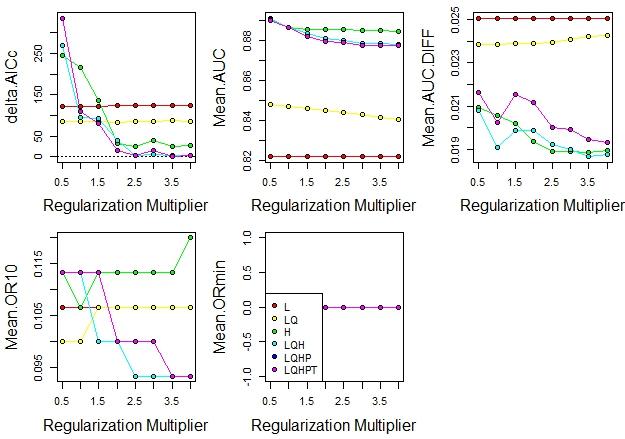


**S10 Fig.** **ENMeval outputs for KUCG with the pre-monsoon occurrence data.**  Model includes environmental covariates: distance to river mouths, distance to land and bathymetric depth.


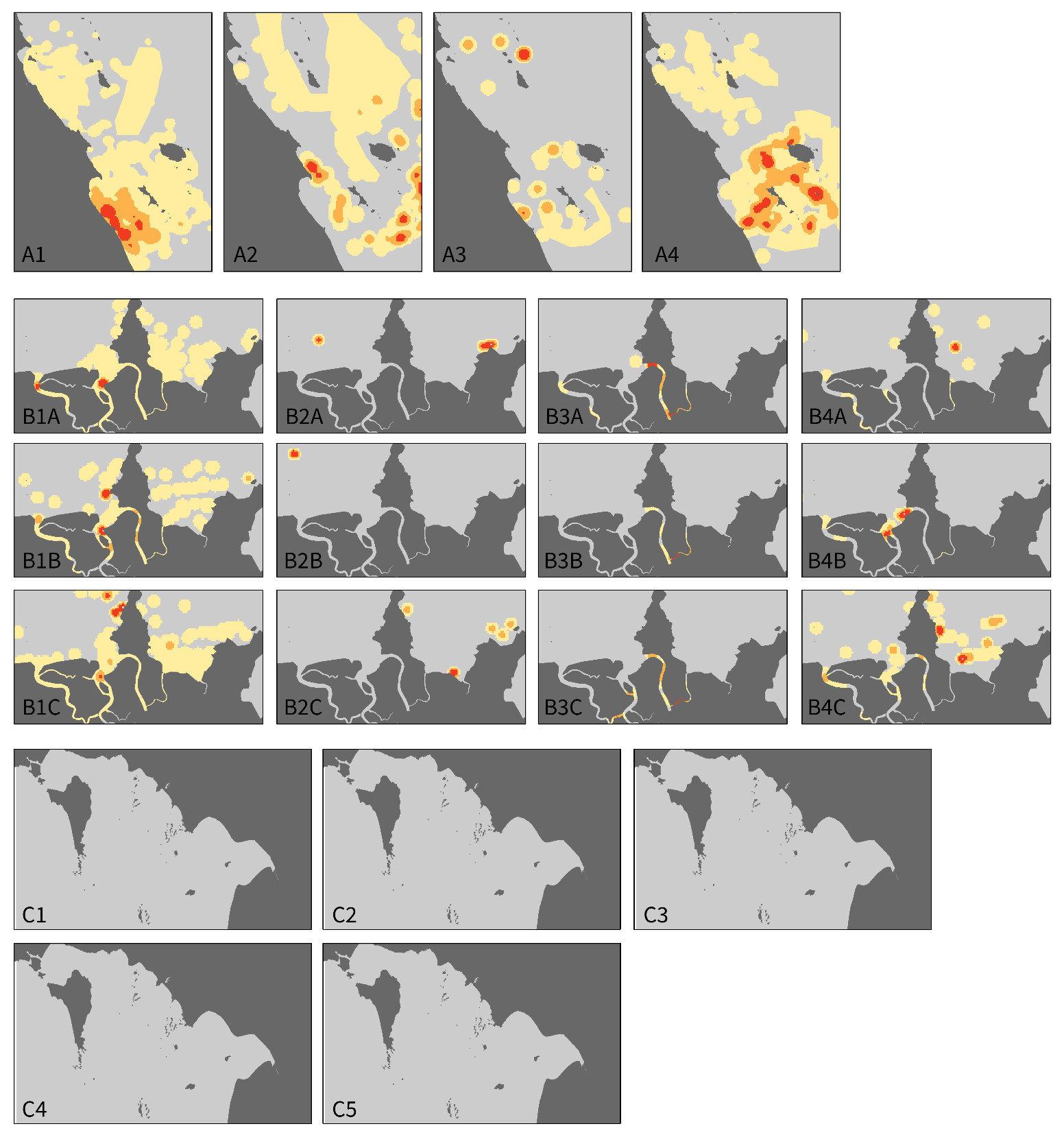

**S11 Fig.** **Spatially explicit layers for ‘intensity of gear use’ criterion by field site.** (A) SBTI and (B) KUCG; fishing gear type: (1) nets, (2) trawls, (3) pots and traps, (4) hook and line, and (5) longlines; and season: (A) dry, (B) pre monsoon, and (C) post monsoon. Warmer colors indicate ratings that contribute to higher exposure scores.


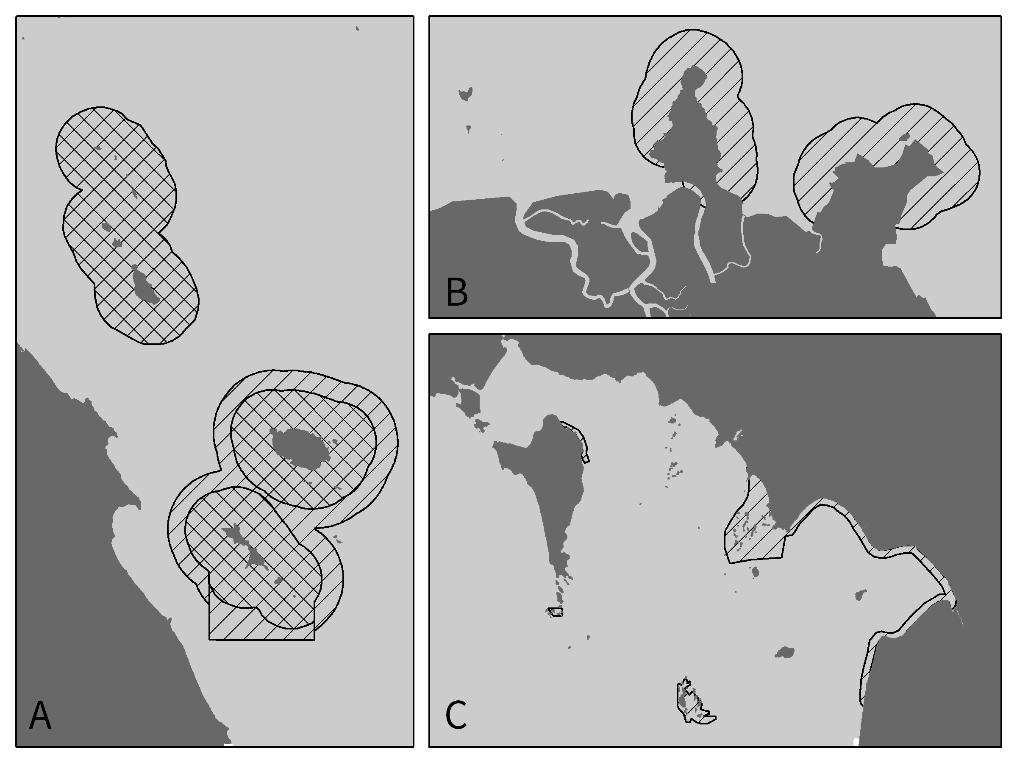

**S12 Fig.** **Layers used to build spatially explicit layer for ‘current status of management’ criterion by field site. (**A) SBTI, (B) KUCG, and (C) KGBR. Cross-hatching indicates areas where management has been identified and implemented (score = 1), hatching indicates areas where management has been identified but not implemented (score = 2), and areas with no hatching indicate that no management has been identified (score = 3).


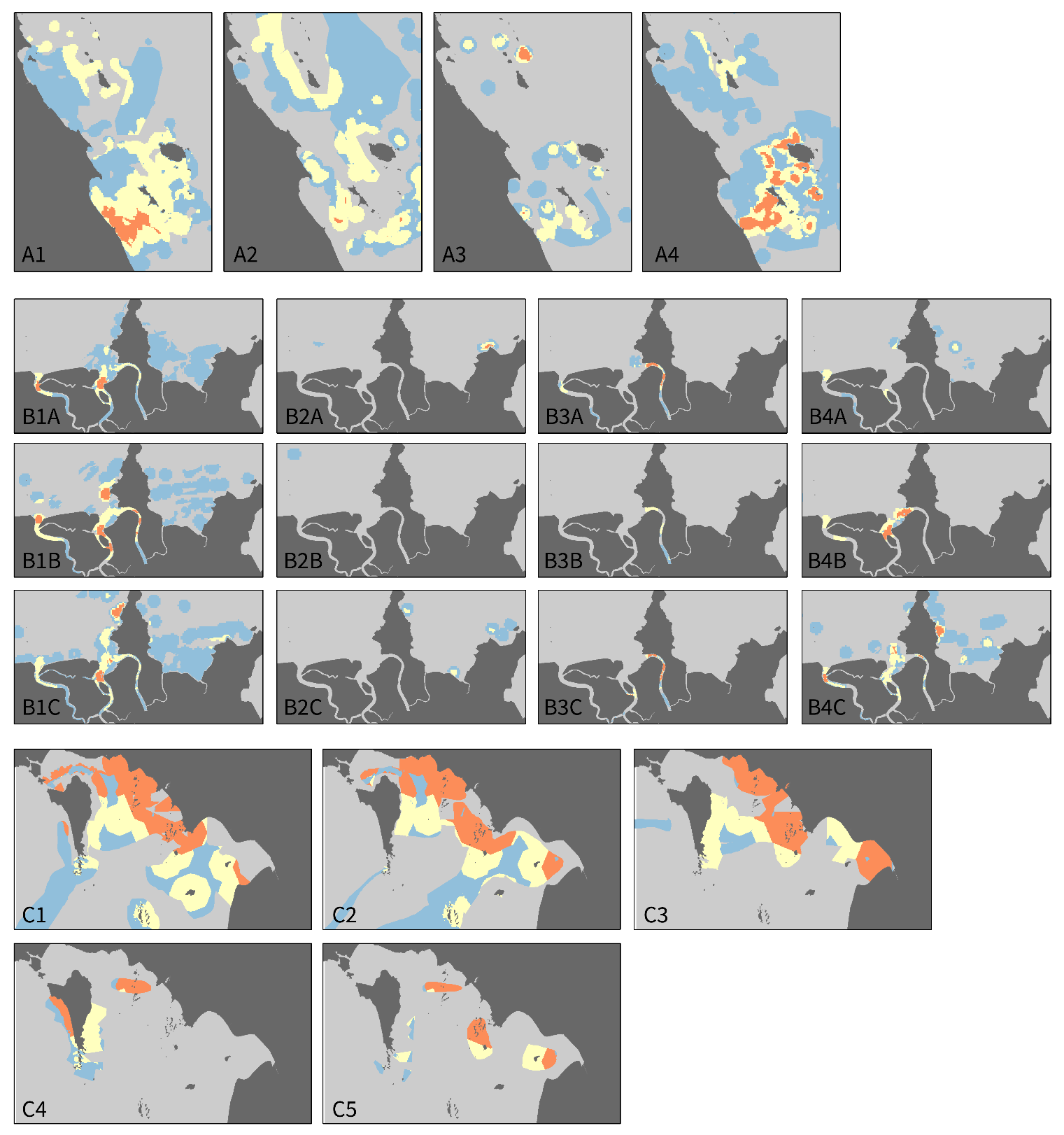

**S13 Fig.** **Spatially explicit layers for ‘likelihood of interaction between gear and species’ criterion by field site.** (A) SBTI, (B) KUCG, and (C) KGBR; fishing gear type: (1) nets, (2) trawls, (3) pots and traps, (4) hook and line, and (5) longlines; and season: (A) dry, (B) pre monsoon, and (C) post monsoon. Warmer colors indicate ratings that contribute to higher exposure scores.
